# Cytoreductive surgery (CRS) with hyperthermic intraoperative peritoneal chemotherapy (HIPEC) versus standard of care (SoC) in people with peritoneal metastases from colorectal, ovarian or gastric origin: protocol for a systematic review and individual participant data (IPD) meta-analyses of effectiveness and cost-effectiveness

**DOI:** 10.1136/bmjopen-2020-039314

**Published:** 2020-05-12

**Authors:** Kurinchi Gurusamy, Claire L Vale, Elena Pizzo, R Bhanot, Brian R Davidson, Tim Mould, Muntzer Mughal, Mark Saunders, Omer Aziz, Sarah O'Dwyer

**Affiliations:** 1 Division of Surgery and Interventional Science, University College London, London, UK; 2 Meta-analysis Group, MRC Clinical Trials Unit at UCL, London, UK; 3 Department of Applied Health Research, University College London, London, UK; 4 Department of HPB Surgery, Royal Free London NHS Foundation Trust, London, UK; 5 Gynaecological Oncology, University College London Hospitals NHS Trust, London, UK; 6 Surgery, University College London Hospital NHS Foundation Trust, London, UK; 7 Colorectal and Peritoneal Oncology Centre, The Christie NHS Foundation Trust, Manchester, UK

**Keywords:** health economics, chemotherapy, qualitative research, oncology, surgery

## Abstract

**Introduction:**

There is uncertainty about whether cytoreductive surgery (CRS)+hyperthermic intraoperative peritoneal chemotherapy (HIPEC) improves survival and/or quality of life compared with standard of care (SoC) in people with peritoneal metastases who can withstand major surgery.

**Primary objectives:**

To compare the relative benefits and harms of CRS+HIPEC versus SoC in people with peritoneal metastases from colorectal, ovarian or gastric cancers eligible to undergo CRS+HIPEC by a systematic review and individual participant data (IPD) meta-analysis.

**Secondary objectives:**

To compare the cost-effectiveness of CRS+HIPEC versus SoC from a National Health Service (NHS) and personal social services perspective using a model-based cost–utility analysis.

**Methods and analysis:**

We will perform a systematic review of literature by updating the searches from MEDLINE, Embase, Cochrane library, Science Citation Index as well as trial registers. Two members of our team will independently screen the search results and identify randomised controlled trials comparing CRS+HIPEC versus SoC for inclusion based on full texts for articles shortlisted during screening. We will assess the risk of bias in the trials and obtain data related to baseline prognostic characteristics, details of intervention and control, and outcome data related to overall survival, disease progression, health-related quality of life, treatment related complications and resource utilisation data. Using IPD, we will perform a two-step IPD, that is, calculate the adjusted effect estimate from each included study and then perform a random-effects model meta-analysis. We will perform various subgroup analyses, meta-regression and sensitivity analyses. We will also perform a model-based cost–utility analysis to assess whether CRS+HIPEC is cost-effective in the NHS setting.

**Ethics and dissemination:**

This project was approved by the UCL Research Ethics Committee (Ethics number: 16023/001). We aim to present the findings at appropriate international meetings and publish the review, irrespective of the findings, in a peer-reviewed journal.

**PROSPERO registration number:**

CRD42019130504.

**Strengths and limitations of this study**

- This study uses individual participant data, which has several advantages over collecting summary data.
- This study will identify the uncertainty of evidence and help in the identification of whether further research is necessary on the topic and how future research should be performed.
- Outcome data may not be available from the randomised controlled trials, even if they are important to patients and healthcare professionals.

## Background and rationale

### What is the problem being addressed?

Approximately 7 million people worldwide and 160 000 people in UK develop colorectal, ovarian or gastric cancer each year,^1^ of whom 8%–50% develop peritoneal metastases. The peritoneum is one of the most common sites of metastases from these cancers^2–8^ and is often the only site of metastases.^7–9^ In general, people with peritoneal metastases have poorer prognosis than those with other metastases (liver or lung),^10^ with median reported survival ranging from 6 months to 24 months depending on from the primary cancers and treatment received.^11–13^

### Treatment of peritoneal metastases

The current standard of care (SoC) of people with peritoneal metastases from these cancers is systemic chemotherapy alone or in combination with either cytoreductive surgery (CRS) or palliative surgery.^7 8 12–15^ CRS+hyperthermic intraoperative peritoneal chemotherapy (HIPEC) is an alternative treatment for these patients. The main principle of CRS+HIPEC is to remove all visible (macroscopic) peritoneal metastases followed by HIPEC to treat any remaining microscopic peritoneal metastases.^16^ HIPEC involves peritoneal circulation of chemotherapy drugs (usually mitomycin C, 5-fluorouracil and oxaliplatin or cisplatin)^17^ heated to temperatures of 42°C, at which the chemotherapy drugs are potentiated.^18^ Until only a decade ago, less than 5% of patients with peritoneal metastases underwent CRS+HIPEC; however, this has progressively increased to about 10% of patients by 2012.^8 9 14^ CRS+HIPEC has been commissioned by National Health Service (NHS) England for patients with peritoneal metastases from appendiceal tumours and colorectal adenocarcinoma.

### Why is this research important to patients and health and care services?

Although CRS+HIPEC has the potential to improve the survival and health-related quality of life (HRQoL) in people with peritoneal metastases,^14 19 20^ there have been concerns raised about its safety. Reports have shown a 30-day mortality after CRS+HIPEC of 1%–3%^6^ and a major complication rate of 32%,^6 21^ although that local audit data from high volume centres suggest that mortality and morbidity rates are somewhat less than in these reports (local audit data). The average costs of CRS+HIPEC per patient varies from about US$20 000–US$80 000.^22–28^ Because of these reasons, this research is important to address the significant uncertainty about the benefits of an intervention that carries significant risk of harm to patients and costs to the NHS.

### Review of existing evidence

There have been several overviews, systematic reviews and health technology assessments (HTAs) investigating this area. Sixteen systematic reviews of comparative studies have been undertaken, comparing CRS+HIPEC to other treatment modalities in peritoneal metastases from colorectal, ovarian or gastric cancer.^6 17 20 29–41^ Ten of these included at least one randomised controlled trial (RCT), but the conclusions were largely based on non-randomised studies.^6 17 20 29 31–33 35 39 41^ Although most of these systematic reviews concluded that CRS+HIPEC can improve survival in people with peritoneal metastases, all had limitations and deficiencies. First, all are at high risk of bias according to risk of bias in systematic reviews tool^42^ with concern about bias across all domains. Second, the systematic reviews included only a single RCT^13^ and/or based their evidence predominantly on non-randomised studies, without any adjustment for baseline differences in disease-related or patient-related prognostic characteristics.^6 17 20 29 31–33 35 39 41^ Finally, meta-analyses could only include a small proportion of the results from the studies because of the way these results had been reported (eg, proportion survived vs median survival).^17 20 29 35 37^ Therefore, there is still considerable uncertainty about the benefits of CRS+HIPEC and which patient groups will benefit from it.

There have been two formal HTAs on this issue.^26 43^ The HTA reviewing patients with peritoneal disease from colorectal cancer concluded that there was moderate quality evidence that CRS+HIPEC prolonged survival based on a single RCT, but the costs were high.^26^ The HTA on ovarian cancer (which did not include any RCTs) concluded there was no clear benefit of CRS+HIPEC for ovarian peritoneal metastases.^43^

### Justification for individual participant data (IPD)

Through the collection and reanalysis of IPD from all relevant RCTs, we aim to overcome the limitations of the existing evidence and provide the highest quality evidence synthesis of the benefits and harms of CRS+HIPEC in patients with peritoneal metastases to inform clinical practice and future research. Importantly, the main advantages of using IPD over aggregate data in this setting are the following.

1. Overcome lack of reporting of key survival outcomes: the key survival outcomes have not been reported in a format that can be meta-analysed. This can be overcome with IPD.
2. Harmonise definitions of performance indicators and outcome: use of IPD can ensure that the definitions of the prognostic and confounding factors, and outcomes are harmonised.
3. Improve the quality of the analysis: IPD is commonly reported to improve the quality of analyses.^44 45^
4. Investigate whether any patient-related or disease-related characteristics impact on the treatment effect at the individual level.

### Aims and objectives

The overarching aim of this project is to answer the following research question:

Does CRS+HIPEC improve survival and/or quality of life compared with SoC in people with peritoneal metastases (from colorectal, ovarian or gastric cancers) who can withstand major surgery and is it cost-effective in the NHS setting?

#### Primary objectives

To compare the relative benefits and harms of CRS+HIPEC versus SoC in people with peritoneal metastases from colorectal, ovarian or gastric cancers eligible to undergo CRS+HIPEC by a systematic review and IPD meta-analysis.

#### Secondary objectives

To compare the cost-effectiveness of CRS+HIPEC versus SoC from an NHS and personal social services (PSS) perspective using a model-based cost–utility analysis.

## General methods

### Eligibility criteria

#### Type of studies

All RCTs regardless of the publication status, year of publication and language of publication will be included.

#### Setting

Secondary or tertiary care with expertise to perform CRS+HIPEC

#### Type of participants

##### Inclusion criteria

People with synchronous or metachronous peritoneal metastases from colorectal cancer, ovarian cancer or gastric cancer, eligible to undergo CRS+HIPEC regardless of the involvement of other organs and whether the primary cancer was resected completely (ie, R0 resection). We will also include people with appendiceal adenocarcinomas under colorectal cancer as they behave in a similar way to colorectal adenocarcinomas.

##### Exclusion criteria

Studies on pseudomyxoma peritonei will be excluded.

#### Intervention

CRS+HIPEC.

#### Control

SoC.

#### Outcomes

##### Primary outcomes

1. Overall survival, defined as time from randomisation until death by any cause.
2. HRQoL using any validated measure.
3. Serious adverse events or Clavien-Dindo grade III or above.^46 47^

##### Secondary outcomes

1. Time to disease progression: defined as time from randomisation to death in people who died of treatment or disease-related causes, time from randomisation to recurrence in people in whom complete CRS was achieved and time from randomisation to disease progression as defined by Response Evaluation Criteria in Solid Tumors (RECIST) criteria of 20% increase in size of the tumour or appearance of new lesions,^48^ or similar criteria used by authors.
2. Non-serious adverse events or Clavien-Dindo grade I or II.^46 47^
3. Patient-reported outcome measures.

### Search strategy

#### Electronic searches

We will search MEDLINE, Embase, Cochrane library and the Science Citation Index for published trials as well as ClinicalTrials.gov, and WHO ICTRP trial registers for ongoing or unreported studies. The search strategies, which combine the Cochrane sensitivity maximising RCT filter^49^ with a combination of subject headings and free-text terms relating to the interventions and diseases of interest, are provided in online supplementary appendix 1. Searches were updated periodically until October 2019.

10.1136/bmjopen-2020-039314.supp1 Supplementary data

#### Other resources

We will also search the references of all identified studies for additional studies eligible for inclusion. We will also contact the American Society of Peritoneal Surface Malignancies, the Canadian HIPEC Collaborative Group, The Peritoneal Surface Oncology Group International and the study authors who agree to participate in this project for further studies.

### Data collection and management

#### Selection of studies

Two review authors will independently screen the titles and abstracts of all records retrieved and make the final selection based on full text (after translation if required, ie, there will be no language restrictions). We will document the process to enable completion of the Preferred Reporting Items for Systematic Reviews and Meta-Analyses flow chart. We will resolve discrepancies through discussion and arbitration.

#### Data collection

At the study level, we will record the contact details of the study author and the study contact, information required to assess the risk of bias and details of the treatment centres (name and the average number of CRS+HIPEC performed per year). At the participant level, we will collect the following details:

1. Centre at which treated.
2. Patient demographics: age, gender, comorbidities and performance index.
3. Cancer details (including severity).
4. Intervention details.
5. Control details.
6. Follow-up details.
7. Outcome data.
8. Resource utilisation data
  - Operating time.
  - Quantity of blood and blood products transfused.
  - Length of hospital stay (including readmissions).
  - Length of intensive care unit stay.
  - Chemotherapy regimen used in HIPEC and in control group if applicable.
  - Proportion in whom surgery was performed and the nature of surgery in the control group.
  - Additional surgery and other palliative treatments.

These data will be sought for all patients randomised into each trial. Up-to-date follow-up will be requested in order to report on longer term outcomes: the existing ethical approval for the studies usually cover collection of data.

The proposed data format and coding conventions for these data will be developed as part of the project to obtain the EVidencE Review of PEritoneal Tumours (EVERPET)-IPD data dictionary. Transfer guide will be developed as part of the project. Although the aim of the conventions is to facilitate data transfer, they are not essential. Data will be accepted in the format most convenient for the individual trial investigator or data centre; however, all personal identifiers (eg, names) are to be removed before sharing. Data should be transferred by encrypted email or source ftp site. Further details are included in the data transfer guide.

#### Data checking and management

Once trial investigators have agreed to provide the IPD, they will be asked to sign a data transfer agreement that covers the transfer, use and storage of that data. By signing up to the agreement, investigators also declare that they have complied with all laws and regulations relating to the conduct of their studies and the collection of data as part of those studies.

On receipt, data will be cleaned and checked for accuracy, consistency and validity. This will include checks for missing data, randomisation integrity, follow-up and censoring. We will query any anomalies with the study contact to ensure that the data are represented accurately and send a summary of the final dataset from each trial to the study contacts for verification.

Once checked and verified, we will store the trial data securely. Access to the data will be restricted to the Project Management Group, who are all trained in data protection and personal data confidentiality and who will act as custodians of the data under the terms of the data transfer agreement, which will be developed as part of this project. In line with that agreement, data will be deposited in the EVERPET-IPD repository.

#### Assessment of risk of bias in included studies

We will use the Cochrane risk of bias tool version 2 to assess the risk of bias in RCTs.^50^

### Meta-analysis of clinical effectiveness

#### Measures of treatment effect and data synthesis

We will use risk ratio for binary outcomes (proportion of people with serious and non-serious adverse events), mean difference (if same scales are reported in the studies) or standardised mean difference (if different scales are reported in the studies) for continuous outcomes (HRQoL), rate ratios for count outcomes (number of serious and non-serious adverse events) and HR for time-to-event outcomes (overall all-cause mortality and time to progression) with their respective 95% CIs.

We will perform a two-step IPD, that is, calculate the adjusted effect estimate from each included study and then perform a random-effects model meta-analysis using DerSimonian and Laird method^51^ for binary outcomes and inverse variance method for other types of outcomes. The reason for choosing the two-step IPD over one-step IPD is the way the confounding factors are reported in the studies, for example, comorbidities can be reported as different types of performance indices and the extent of peritoneal disease can be reported in different ways.^52 53^ However, if we agree on an approximation to convert different performance indices into a single measure and convert the different measures of extent of peritoneal involvement into a single measure, we will perform a single-step meta-analysis to check the robustness of the two-step meta-analysis. We will test our assumptions in approximations (of the different performance indices into a single measure and different measures of extent of peritoneal involvement into a single measure) by sensitivity analyses. We will use multilevel modelling to take the clustering of data in the studies into account for the one-step IPD meta-analysis, as the unit of analysis will be the individual participant.

#### Dealing with missing data

We will perform an intention-to-treat analysis whenever possible.^54^ If data on the classification of the treatment as intervention or control is missing and cannot be ascertained though discussion with triallists, we will exclude such participants. If outcome data are missing, we will use multiple imputation method if the data are likely to be missing at random or best-case and worst-case scenarios analysis if it is felt that the outcome data are not missing at random.

#### Assessment and investigation of heterogeneity

We will assess the clinical and methodological heterogeneity by carefully examining the characteristics and design of included trials. Clinical heterogeneity could be due to the type of participants included in the studies (performance index, stage of cancer, extent of peritoneal involvement and other organ involvement), different interventions (complete CRS or not and chemotherapy agents used), different controls (chemotherapy alone or CRS or both), whether complete CRS was achieved (if the control group was CRS) or different follow-up methods (routine imaging vs clinical examination). Different study designs and risk of bias may contribute to methodological heterogeneity. We will calculate and report the between-trial SD and I^2^ as measures of heterogeneity.

If we identify substantial clinical, methodological or statistical heterogeneity, we will explore and address it in subgroup analyses and/or meta-regression using participant level covariates on the sources of clinical heterogeneity mentioned above except for routine imaging that will be a trial-level covariate. All sources of methodological heterogeneity will be trial-level covariates.

#### Sensitivity analysis

We will perform the following sensitivity analyses to assess the impact of:

- Data not missing at random.
- Non-participation in the IPD.
- Methods (two-step vs single-step) and model (fixed-effect vs random-effects model) used for meta-analysis.
- Using ‘time from diagnosis’ rather than ‘time from randomisation’ for defining ‘time to disease progression’.
- Risk of bias.

#### Network meta-analysis

We will also perform a network meta-analysis of aggregate data to compare the three treatments:

1. CRS+HIPEC+systemic chemotherapy.
2. CRS alone+systemic chemotherapy.
3. Systemic chemotherapy.

#### Reporting bias

We will assess reporting bias by the completeness of search.

#### Confidence in results

The uncertainty in results will be evaluated using the Grading of Recommendations Assessment, Development and Evaluation (GRADE) methodology.^55^

### Cost-effectiveness analysis

We will follow the National Institute for Health and Care Excellence (NICE) methodological standards for conducting our cost-effectiveness analysis.^56^

#### Model

We will perform a model-based cost–utility analysis estimating mean costs and quality-adjusted life years (QALYs) per patient. We will compare CRS+HIPEC versus SoC in each of the three cancers by three separate cost-effectiveness analyses. The time horizon will be lifetime time horizon. We will calculate the costs from the NHS and PSS perspective. We will discount the costs and utilities at the rate of 3.5% per annum.^56^

We will create a decision tree model (one for each cancer) along the lines of the model that we used to compare two types of surgeries in pancreatic cancer.^57^ Briefly, a patient with peritoneal metastases from one of the three cancers (colorectal cancer, ovarian cancer or gastric cancer) and eligible for CRS+HIPEC can either undergo CRS+HIPEC or SoC. A proportion of patients undergoing CRS+HIPEC will have complete CRS (ie, all macroscopic tumour is removed). A proportion of patients in whom CRS+HIPEC will develop complications (whether complete CRS was achieved or not), a proportion of whom may die within 90 days. Those who are alive at 90 days may die subsequently (a Markov model will be used to model this). The decision tree pathways in the people who had SoC will be identical: some will have complete CRS, some will have complications, some will die within 90 days and some will die after 90 days.

Most of the information required for populating the decision tree (including resource utilisation data) will be obtained from the systematic review and IPD meta-analysis. For information not available from the systematic review and IPD meta-analysis, we will perform literature searches of NHS Economic Evaluation Database, the Health Economic Evaluations Database, MEDLINE and Embase (for MEDLINE and Embase, we will combine the search strategy from online supplementary appendix 1 with sensitivity maximising ‘economics’ filter developed as a part of The Hedges Project of the Health Information Research Unit of McMaster University). We will also review the Cost-Effectiveness Analysis Registry at Tufts University for information on quality of life. Currently, there is no Healthcare Resource Group code available for CRS+HIPEC and SoC (which will vary according to the nature of the treatment). We will obtain resource utilisation data as part of the systematic review and IPD (please see above) and convert these to costs on the basis of NHS National Tariff, NHS National Schedule of Reference costs, British National Formulary and/or local estimates as required.

We will assume that the people who die in each period will do so at a constant rate during the period and check whether this assumption is true using the IPD. If this assumption is not true, then we will use more complex models to mirror the survival curves based on the IPD. When no data are available from the IPD or published sources, a range of values will be used in the model. We will tabulate the inputs used in the decision tree model and the source of these inputs in the project report.

#### Measuring cost-effectiveness

We will measure cost-effectiveness using net monetary benefits (NMBs). For each treatment, we will calculate the NMB as the mean QALYs per patient accruing to that treatment multiplied by decision makers’ maximum willingness to pay for a QALY (also referred to as the cost-effectiveness threshold), minus the mean cost per patient for the treatment. In the UK, the upper limit of the maximum willingness to pay for a QALY is £20 000–£30 000.^56^ NMBs will be calculated using the base case parameter values to obtain the deterministic results, which do not depend on chance. The option with the highest NMB represents best value for money. The NMB for CRS+HIPEC minus the NMB for SoC is the incremental NMB. If the incremental NMB is positive, then CRS+HIPEC represents better value for money; if it is negative, the SoC represents better value for money.

A probabilistic sensitivity analysis (PSA) will also be undertaken.^56^ The PSA involves Monte Carlo simulation and takes variability of all selected inputs into account simultaneously. Distributions will be assigned to parameters to reflect the uncertainty with each parameter value. A random value from the corresponding distribution for each parameter will be selected (by the computer). This will generate an estimate of the mean cost and mean QALYs and the NMB associated with each treatment. This will be repeated 10 000 times, and the results for each simulation will be noted. The mean costs, QALYs and NMB for each treatment will be calculated from the 10 000 simulations; these are probabilistic results because they depend on chance. We will increase the simulations as required by a stability test, which involves ensuring that the SE when the PSA is repeated 30 times is within 2% of each other. The NMB will also be calculated for each of the 10 000 simulations, and the proportion of times each treatment had the highest NMB will be calculated for a range of values for the maximum willingness to pay for a QALY. These will be summarised graphically using cost-effectiveness acceptability curves. We will derive the 95% CIs around the base case values using the 2.5 and 97.5 percentiles calculated from the PSA. We will also perform a value of information analysis and calculate the expected value of perfect information and the expected value of partially perfect information.

For the deterministic univariate sensitivity analysis, each variable in the cost-effectiveness model will be varied one at a time. The results of the sensitivity analysis will be represented in the tornado diagram that reflects the variation in the NMB within the range of the lowest and highest value used for a parameter with all else equal. If the variation in the NMB includes 0, then there is uncertainty in the cost-effectiveness due to the variation of the parameter.

We will also perform various subgroup analyses guided by the results of the systematic reviews and IPD meta-analyses but will include subgroup analysis of different types of control (ie, CRS alone or systemic chemotherapy alone or both) as a minimum. We will also perform a sensitivity analysis using information from ‘real-life’ prospective data from Christie NHS Foundation Trust (and from other NHS specialist centres if such information is available).

We will follow the ‘Consolidated Health Economic Evaluation Reporting Standards’ reporting checklist for reporting the cost-effectiveness analysis.^58^

## Patient and public involvement

A patient representative was involved in the preparation of this grant proposal and found that this research proposal was important to patients. Additional patient representatives will be identified. A patient representative will also be part of the research oversight committee.

## Supplementary Material

Reviewer comments

Author's manuscript
