# Supplementary material for: Cytoreductive surgery (CRS) with hyperthermic intraoperative peritoneal chemotherapy (HIPEC) versus standard of care (SoC) in people with peritoneal metastases from colorectal, ovarian or gastric origin: protocol for a systematic review and individual participant data (IPD) meta-analyses of effectiveness and cost-effectiveness
Source: BMJ Open. 2020 May 12;10(5):e039314. doi: 10.1136/bmjopen-2020-039314 (PMC7228534; doi:10.1136/bmjopen-2020-039314)
Supplement: Supplementary data [file bmjopen-2020-039314supp001.pdf]

Appendix 1: Search Strategies

Medline

1. Hyperthermia, Induced/

2. ((hyperthermic or heated) adj3 (intraperitoneal or intra-peritoneal) adj3 (chemotherapy or chemotherapies)).ti,ab.

3. (intraperitoneal adj3 chemohyperthermia).ti,ab.

4. (HIPEC or IPHC or HIIC).ti,ab.

5. 1 or 2 or 3 or 4

6. Cytoreduction Surgical Procedures/

7. ((cytoreductive or cytoreduction or debulking) adj3 (surgery or surgeries or surgical or procedure or procedures)).ti,ab.

8. 6 or 7

9. 5 or 8

10. exp Colorectal Neoplasms/

11. exp Ovarian Neoplasms/

12. Stomach Neoplasms/

13. ((colorectal or bowel or colon or colonic or rectum or rectal or ovary or ovaries or ovarian or gastric or stomach) adj3 (cancer or cancers or carcinoma or carcinomas or tumour or tumours or tumor or tumors or neoplasm or neoplasms)).ti,ab.

14. 10 or 11 or 12 or 13

15. 9 and 14

16. randomized controlled trial.pt.

17. controlled clinical trial.pt.

18. randomized.ab.

19. placebo.ab.

20. drug therapy.fs.

21. randomly.ab.

22. trial.ab.

23. groups.ab.

24. 16 or 17 or 18 or 19 or 20 or 21 or 22 or 23

25. exp animals/ not humans.sh.

26. 24 not 25

27. 15 and 26

28. (cost: or cost benefit analys: or health care costs).mp.

29. 15 and 28

30. 27 or 29

Embase

1. hyperthermic intraperitoneal chemotherapy/

2. ((hyperthermic or heated) adj3 (intraperitoneal or intra-peritoneal) adj3 (chemotherapy or chemotherapies)).ti,ab.

3. (intraperitoneal adj3 chemohyperthermia).ti,ab.

4. (HIPEC or IPHC or HIIC).ti,ab.

5. 1 or 2 or 3 or 4

6. cytoreductive surgery/

7. ((cytoreductive or cytoreduction or debulking) adj3 (surgery or surgeries or surgical or procedure or procedures)).ti,ab.

8. 6 or 7

9. 5 or 8

10. exp colon cancer/

11. exp rectum cancer/

12. exp ovary cancer/

13. exp stomach cancer/

14. ((colorectal or bowel or colon or colonic or rectum or rectal or ovary or ovaries or ovarian or gastric or stomach) adj3 (cancer or cancers or carcinoma or carcinomas or tumour or tumours or tumor or tumors or neoplasm or neoplasms)).ti,ab.

15. 10 or 11 or 12 or 13 or 14

16. 9 and 15

17. exp crossover-procedure/ or exp double-blind procedure/ or exp randomized controlled trial/ or single-blind procedure/

18. (((((random* or factorial* or crossover* or cross over* or cross-over* or placebo* or double*) adj blind*) or single*) adj blind*) or assign* or allocat* or volunteer*).af.

19. 17 or 18

20. 16 and 19

21. (cost or costs).tw.

22. 16 and 21

23. 20 or 22

Cochrane

#1 MeSH descriptor: [Hyperthermia, Induced] this term only

#2 ((hyperthermic or heated) near/3 (intraperitoneal or intra-peritoneal) near/3 (chemotherapy or chemotherapies))

#3 (intraperitoneal near/3 chemohyperthermia)

#4 (HIPEC or IPHC or HIIC)

#5 #1 or #2 or #3 or #4

#6 MeSH descriptor: [Cytoreduction Surgical Procedures] this term only

#7 ((cytoreductive or cytoreduction or debulking) near/3 (surgery or surgeries or surgical or procedure or procedures))

#8 #6 or #7

#9 #5 or #8

#10 MeSH descriptor: [Colorectal Neoplasms] explode all trees

#11 MeSH descriptor: [Ovarian Neoplasms] explode all trees

#12 MeSH descriptor: [Stomach Neoplasms] this term only

#13 ((colorectal or bowel or colon or colonic or rectum or rectal or ovary or ovaries or ovarian or gastric or stomach) near/3 (cancer or cancers or carcinoma or carcinomas or tumour or tumours or tumor or tumors or neoplasm or neoplasms))

#14 #10 or #11 or #12 or #13

#15 #9 and #14

Science Citation Index

1 TS=((hyperthermic or heated) near/3 (intraperitoneal or intra-peritoneal) near/3 (chemotherapy or chemotherapies))

2 TS=(intraperitoneal near/3 chemohyperthermia)

3 TS=(HIPEC or IPHC or HIIC)

4 #3 OR #2 OR #1

5 TS=((cytoreductive or cytoreduction or debulking) near/3 (surgery or surger-ies or surgical or procedure or procedures))

6 #5 or #4

7 TS=((colorectal or bowel or colon or colonic or rectum or rectal or ovary or ovaries or ovarian or gastric or stomach) near/3 (cancer or cancers or carci-noma or carcinomas or tumour or tumours or tumor or tumors or neoplasm or neoplasms))

#8 TS=(random* or placebo* or blind* or meta-analysis or cost or costs)

#9 #8 AND #7 AND #6

WHO trials register

Condition: colorectal OR bowel OR colon OR colonic OR rectum OR rectal OR ovary OR ovaries OR ovarian OR gastric OR stomach

Intervention: HIPEC OR hyperthermic intraperitoneal chemotherapy OR IPHC OR intraperitoneal chemohyperthermia OR HIIC OR heated intraoperative intraperitoneal chemotherapy OR cytoreductive surgery OR CRS

ClinicalTrials.gov

Condition: colorectal OR bowel OR colon OR colonic OR rectum OR rectal OR ovary OR ovaries OR ovarian OR gastric OR stomach

Study Type: Interventional Studies (Clinical Trials)

Intervention/treatment: HIPEC OR hyperthermic intraperitoneal chemotherapy OR IPHC OR intraperitoneal chemohyperthermia OR HIIC OR heated intraoperative intraperitoneal chemotherapy OR cytoreductive surgery OR CRS

Interventional studies, phase 2,3,4

Interventional Studies | colorectal OR bowel OR colon OR colonic OR rectum OR rectal OR ovary OR ovaries OR ovarian OR gastric OR stomach | HIPEC OR hyperthermic intraperitoneal chemotherapy OR IPHC OR intraperitoneal chemohyperthermia OR HIIC OR heated intraoperative intraperitoneal chemotherapy OR cytoreductive surgery OR CRS | Phase 2, 3, 4

Cost-Effectiveness Analysis (CEA) Registry

The following terms were searched:

Hyperthermic

Cytoreduction

Cytoreductive
