# Supplementary material for: Cytoreductive surgery (CRS) with hyperthermic intraoperative peritoneal chemotherapy (HIPEC) versus standard of care (SoC) in people with peritoneal metastases from colorectal, ovarian or gastric origin: protocol for a systematic review and individual participant data (IPD) meta-analyses of effectiveness and cost-effectiveness
Source: BMJ Open. 2020 May 12;10(5):e039314. doi: 10.1136/bmjopen-2020-039314 (PMC7228534; doi:10.1136/bmjopen-2020-039314)
Supplement: Author's manuscript [file bmjopen-2020-039314.draft_revisions.pdf]

BMJ Open

BMJ Open is committed to open peer review. As part of this commitment we make the peer review history of every article we publish publicly available.

When an article is published we post the peer reviewers' comments and the authors' responses online. We also post the versions of the paper that were used during peer review. These are the versions that the peer review comments apply to.

The versions of the paper that follow are the versions that were submitted during the peer review process. They are not the versions of record or the final published versions. They should not be cited or distributed as the published version of this manuscript.

BMJ Open is an open access journal and the full, final, typeset and author-corrected version of record of the manuscript is available on our site with no access controls, subscription charges or pay-per-view fees (http://bmjopen.bmj.com).

If you have any questions on BMJ Open's open peer review process please email info.bmjopen@bmj.com

BMJ Open

Cytoreductive surgery (CRS) with hyperthermic intraoperative peritoneal chemotherapy (HIPEC) versus standard of care (SoC) in people with peritoneal metastases from colorectal, ovarian or gastric origin: protocol for a systematic review and individual participant data (IPD) meta-analyses of effectiveness and cost-effectiveness

Journal:	*BMJ Open*
Manuscript ID	bmjopen-2020-039314
Article Type:	Protocol
Date Submitted by the Author:	14-Apr-2020

Complete List of Authors:	Gurusamy, Kurinchi; University College London,<br>Vale, Claire; MRC Clinical Trials Unit at UCL, Meta-analysis Group<br>Pizzo, Elena; University College London, Applied Health Research<br>Bhanot, R; University College London, Patient representative (undisclosed)<br>Davidson, Brian; Royal Free London NHS Foundation Trust, HPB Surgery<br>Mould, Tim; University College London Hospitals NHS Foundation Trust<br>Mughal, Muntzer; University College London Hospitals NHS Foundation Trust,<br>Saunders, Mark ; The Christie NHS Foundation Trust,<br>Aziz, Omer; The Christie NHS Foundation Trust<br>O'Dwyer, Sarah; The Christie NHS Foundation Trust

Keywords:	HEALTH ECONOMICS, CHEMOTHERAPY, QUALITATIVE RESEARCH, ONCOLOGY

SCHOLARONE™
Manuscripts

I, the Submitting Author has the right to grant and does grant on behalf of all authors of the Work (as defined in the below author licence), an exclusive licence and/or a non-exclusive licence for contributions from authors who are: i) UK Crown employees; ii) where BMJ has agreed a CC-BY licence shall apply, and/or iii) in accordance with the terms applicable for US Federal Government officers or employees acting as part of their official duties; on a worldwide, perpetual, irrevocable, royalty-free basis to BMJ Publishing Group Ltd ("BMJ") its licensees and where the relevant Journal is co-owned by BMJ to the co-owners of the Journal, to publish the Work in this journal and any other BMJ products and to exploit all rights, as set out in our licence.

The Submitting Author accepts and understands that any supply made under these terms is made by BMJ to the Submitting Author unless you are acting as an employee on behalf of your employer or a postgraduate student of an affiliated institution which is paying any applicable article publishing charge ("APC") for Open Access articles. Where the Submitting Author wishes to make the Work available on an Open Access basis (and intends to pay the relevant APC), the terms of reuse of such Open Access shall be governed by a Creative Commons licence – details of these licences and which Creative Commons licence will apply to this Work are set out in our licence referred to above.

Other than as permitted in any relevant BMJ Author's Self Archiving Policies, I confirm this Work has not been accepted for publication elsewhere, is not being considered for publication elsewhere and does not duplicate material already published. I confirm all authors consent to publication of this Work and authorise the granting of this licence.

For peer review only

Cytoreductive surgery (CRS) with hyperthermic intraoperative peritoneal chemotherapy (HIPEC) versus standard of care (SoC) in people with peritoneal metastases from colorectal, ovarian or gastric origin: protocol for a systematic review and individual participant data (IPD) meta-analyses of effectiveness and cost-effectiveness

Prof Kurinchi Gurusamy PhD[1], Dr Claire Vale PhD[2], Dr Elena Pizzo PhD[3], Mr R Bhanot BSc[1], Prof Brian Davidson MD FRCS[1], Mr Tim Mould DM FRCOG[4], Prof Muntzer Mughal FRCS[1], Dr Mark Saunders PhD[5], Mr Omer Aziz PhD[5], Prof Sarah O'Dwyer MD FRCS[5]

[1] Division of Surgery and Interventional Science, University College London, London

[2] MRC trials Unit, University College London

[3] Centre of Applied Health Research, University College London

[4] University College London NHS Foundation Trust

[5] The Christie NHS Foundation Trust

Corresponding author: Prof Kurinchi Gurusamy

Division of Surgery and Interventional Science, University College London

Hampstead Campus, 9th Floor, Royal Free Hospital

Rowland Hill Street

London

NW3 2PF

United Kingdom

Email: k.gurusamy@ucl.ac.uk

Tel: +44 (0)20 8016 8116

For peer review only

Contact details of authors

Kurinchi Gurusamy: Division of Surgery and Interventional Science, University College London, Hampstead Campus, Royal Free Hospital, Rowland Hill Street, London, NW3 2PF, United Kingdom; k.gurusamy@ucl.ac.uk

Claire Vale: MRC trials Unit, University College London, 90 High Holborn, London, WC1V 6LJ; claire.vale@ucl.ac.uk

Elena Pizzo: Centre of Applied Health Research, University College London, 1-19 Torrington Place, London WC1E 7HB; e.pizzo@ucl.ac.uk

R Bhanot: patient representative (first name and email to be disclosed on a need to know basis; address will not be disclosed)

Brian Davidson: Division of Surgery and Interventional Science, University College London, Hampstead Campus, Royal Free Hospital, Rowland Hill Street, London, NW3 2PF, United Kingdom, b.davidson@ucl.ac.uk

Tim Mould: University College London NHS Foundation Trust, 235 Euston Rd, Bloomsbury, London NW1 2BU; t.mould@nhs.net

Muntzer Mughal: Division of Surgery and Interventional Science, University College London, Bloomsbury Campus, Charles Bell House. 43-45 Foley Street, London, W1W 7TS; muntzer.mughal@nhs.net

Mark Saunders: The Christie NHS Foundation Trust, Christie Hospital, Palatine Rd, Withington, Manchester M20 3JJ; Mark.Saunders@christie.nhs.uk

Omer Aziz: Christie Hospital, Palatine Rd, Withington, Manchester M20 3JJ; Omer.Aziz@christie.nhs.uk

Sarah O'Dwyer: Christie Hospital, Palatine Rd, Withington, Manchester M20 3JJ; Sarah.O'Dwyer@christie.nhs.uk

For peer review only

Ethics approval: This project was approved by the UCL Research Ethics Committee (Ethics number: 16023/001).

PROSPERO registration: CRD42019130504

Funding: This study is funded by the National Institute for Health Research (NIHR) HTA programme (HTA - Project: 17/135/02).

Role of funder: The funder sought independent peer review before funding and approved the protocol. All dissemination must be approved by the funder before dissemination.

Disclaimer: The views expressed are those of the author(s) and not necessarily those of the NIHR or the Department of Health and Social Care.

Sponsor: University College London

Disclosures: The clinical practice of the clinicians in the project: Mr Tim Mould, Mr Muntzer Mughal, Dr Mark Saunders, Mr Omer Aziz, and Prof Sarah O'Dwyer may be altered by the findings of the review.

Author contributions: Kurinchi Gurusamy and Claire Vale wrote the manuscript. The manuscript was critically revised by Elena Pizzo, R Bhanot, Brian Davidson, Tim Mould, Muntzer Mughal, Mark Saunders, Omer Aziz, Sarah O'Dwyer. Kurinchi Gurusamy is the guarantor of this manuscript.

Keywords: systematic review, individual participant data, meta-analysis, peritoneal metastases, hyperthermic intraoperative peritoneal chemotherapy

Word count: 3725 words

For peer review only

Abstract

Introduction: There is uncertainty about whether cytoreductive surgery (CRS) + hyperthermic intraoperative peritoneal chemotherapy (HIPEC) improves survival and/or quality of life compared to standard of care (SoC) in people with peritoneal metastases who can withstand major surgery.

Primary objectives: To compare the relative benefits and harms of CRS+HIPEC versus SoC in people with peritoneal metastases from colorectal, ovarian, or gastric cancers eligible to undergo CRS+HIPEC by a systematic review and individual participant data (IPD) meta-analysis.

Secondary objectives: To compare the cost-effectiveness of CRS+HIPEC versus SoC from a National Health Service (NHS) and personal social services (PSS) perspective using a model-based cost-utility analysis.

Methods and analysis: We will perform a systematic review of literature by updating the searches from MEDLINE, EMBASE, Cochrane library, Science Citation Index as well as trial registers. Two members of our team will independently screen the search results and identify randomised controlled trials (RCTs) comparing CRS+HIPEC versus SoC for inclusion based on full texts for articles shortlisted during screening. We will assess the risk of bias in the trials and obtain data related to baseline prognostic characteristics, details of intervention and control, and outcome data related to overall survival, disease progression, health-related quality of life, treatment related complications, and resource utilisation data. Using individual participant data (IPD), we will perform a two-step IPD, i.e. calculate the adjusted effect estimate from each included study and then perform a random-effects model meta-analysis. We will perform various subgroup analyses, metaregression, and sensitivity analyses. We will also perform a model-based cost-utility analysis to assess whether CRS+HIPEC is cost-effective in the NHS setting.

Ethics and Dissemination: This project was approved by the UCL Research Ethics Committee (Ethics number: 16023/001). We aim to present the findings at appropriate international meetings and publish the review, irrespective of the findings, in a peer-reviewed journal.

For peer review only

Article summary

Strengths and limitations of this study

- This study utilises individual participant data, which has several advantages over collecting summary data.
- This study will identify the uncertainty of evidence and help in the identification of whether further research is necessary on the topic and how future research should be performed.
- Outcome data may not be available from the randomised controlled trials, even if they are important to patients and healthcare professionals.

For peer review only

Background and rationale

What is the problem being addressed?

Approximately 7 million people worldwide and 160,000 people in UK develop colorectal, ovarian, or gastric cancer each year [1], of whom 8% to 50% develop peritoneal metastases. The peritoneum is one of the commonest sites of metastases from these cancers [2-8], and is often the only site of metastases [7-9]. In general, people with peritoneal metastases have poorer prognosis than those with other metastases (liver or lung) [10], with median reported survival ranging from 6 to 24 months depending on from the primary cancers and treatment received [11-13].

Treatment of peritoneal metastases

The current standard of care of people with peritoneal metastases from these cancers is systemic chemotherapy alone or in combination with either cytoreductive surgery (CRS) or palliative surgery [7, 8, 12-15]. CRS + hyperthermic intraoperative peritoneal chemotherapy (HIPEC) is an alternative treatment for these patients. The main principle of CRS+HIPEC is to remove all visible (macroscopic) peritoneal metastases followed by HIPEC to treat any remaining microscopic peritoneal metastases [16]. HIPEC involves peritoneal circulation of chemotherapy drugs (usually mitomycin C, 5-Fluorouracil, and oxaliplatin, or cisplatin) [17] heated to temperatures of 42° C, at which the chemotherapy drugs are potentiated [18]. Until only a decade ago, less than 5% of patients with peritoneal metastases underwent CRS+HIPEC, however this has progressively increased to about 10% of patients by 2012 [8, 9, 14]. CRS+HIPEC has been commissioned by NHS England for patients with peritoneal metastases from appendiceal tumours and colorectal adenocarcinoma.

Why is this research important to patients and health and care services?

Although CRS+HIPEC has the potential to improve the survival and health-related quality of life (HRQoL) in people with peritoneal metastases [14, 19, 20], there have been concerns raised about its

For peer review only

safety. Reports have shown a 30-day mortality after CRS+HIPEC of 1-3% [6], and a major complication rate of 32% [6, 21], albeit that local audit data from high volume centres suggest that mortality and morbidity rates are somewhat less than in these reports (local audit data). The average costs of CRS+HIPEC per patient varies from about 20,000 USD to 80,000 USD [22-28]. Because of these reasons, this research is important to address the significant uncertainty about the benefits of an intervention that carries significant risk of harm to patients and costs to the NHS.

Review of existing evidence

There have been several overviews, systematic reviews, and health technology assessments (HTA) investigating this area. Sixteen systematic reviews of comparative studies have been undertaken, comparing CRS+HIPEC to other treatment modalities in peritoneal metastases from colorectal, ovarian, or gastric cancer [6, 17, 20, 29-41]. Ten of these included at least one RCT, but the conclusions were largely based on non-randomised studies [6, 17, 20, 29, 31-33, 35, 39, 41]. Although most of these systematic reviews concluded that CRS+HIPEC can improve survival in people with peritoneal metastases, all had limitations and deficiencies. Firstly, all are at high risk of bias according to ROBIS (risk of bias in systematic reviews) tool [42] with concern about bias across all domains. Secondly, the systematic reviews included only a single RCT [13] and/or based their evidence predominantly on non-randomised studies, without any adjustment for baseline differences in disease-related or patient-related prognostic characteristics [6, 17, 20, 29, 31-33, 35, 39, 41]. Finally, meta-analyses could only include a small proportion of the results from the studies because of the way these results had been reported (e.g. proportion survived versus median survival) [17, 20, 29, 35, 37]. Therefore, there is still considerable uncertainty about the benefits of CRS+HIPEC and which patient groups will benefit from it.

There have been two formal HTAs on this issue [26, 43]. The HTA reviewing patients with peritoneal disease from colorectal cancer concluded that there was moderate quality evidence that CRS+HIPEC prolonged survival based on a single RCT, but the costs were high [26]. The HTA on

For peer review only

ovarian cancer (which did not include any RCTs) concluded there was no clear benefit of CRS+HIPEC for ovarian peritoneal metastases [43].

Justification for IPD

Through the collection and reanalysis of IPD from all relevant randomised controlled trials, we aim to overcome the limitations of the existing evidence and provide the highest quality evidence synthesis of the benefits and harms of CRS + HIPEC in patients with peritoneal metastases to inform clinical practice and future research. Importantly, the main advantages of using IPD over aggregate data in this setting are the following.

1. Overcome lack of reporting of key survival outcomes: The key survival outcomes have not been reported in a format that can be meta-analysed. This can be overcome with IPD.
2. Harmonise definitions of performance indicators and outcome: Use of IPD can ensure that the definitions of the prognostic and confounding factors, and outcomes are harmonised.
3. Improve the quality of the analysis: IPD is commonly reported to improve the quality of analyses [44, 45].
4. Investigate whether any patient-related or disease-related characteristics impact on the treatment effect at the individual level

Aims and objectives

The overarching aim of this project is to answer the following research question:

Does CRS+HIPEC improve survival and/or quality of life compared to SoC in people with peritoneal metastases (from colorectal, ovarian, or gastric cancers) who can withstand major surgery and is it cost-effective in the NHS setting?

Primary objectives

To compare the relative benefits and harms of CRS+HIPEC versus SoC in people with peritoneal metastases from colorectal, ovarian, or gastric cancers eligible to undergo CRS+HIPEC by a systematic review and IPD meta-analysis.

For peer review only

Secondary objectives

To compare the cost-effectiveness of CRS+HIPEC versus SoC from an NHS and PSS (personal social services) perspective using a model-based cost-utility analysis.

General Methods

Eligibility criteria

Type of studies

All RCTs regardless of the publication status, year of publication, and language of publication will be included.

Setting

Secondary or tertiary care with expertise to perform CRS+HIPEC

Type of participants

Inclusion criteria

People with synchronous or metachronous peritoneal metastases from colorectal cancer, ovarian cancer, or gastric cancer, eligible to undergo CRS+HIPEC regardless of the involvement of other organs and whether the primary cancer was resected completely (i.e. R0 resection). We will also include people with appendiceal adenocarcinomas under colorectal cancer as they behave in a similar way to colorectal adenocarcinomas.

Exclusion criteria

Studies on pseudomyxoma peritonei (PMP) will be excluded.

Intervention

CRS+HIPEC

Control

Standard of care (SoC)

For peer review only

Outcomes

Primary outcomes

1. Overall survival, defined as time from randomisation until death by any cause.
2. HRQoL using any validated measure
3. Serious adverse events or Clavien-Dindo grade III or above [46, 47]

Secondary outcomes

4. Time to disease progression: defined as time from randomisation to death in people who died of treatment or disease-related causes, time from randomisation to recurrence in people in whom complete CRS was achieved, and time from randomisation to disease progression as defined by RECIST (Response Evaluation Criteria in Solid Tumors) criteria of 20% increase in size of the tumour or appearance of new lesions [48], or similar criteria used by authors
5. Non-serious adverse events or Clavien-Dindo grade I or II [46, 47]
6. Patient reported outcome measures

Search strategy

Electronic searches

We will search MEDLINE, EMBASE, Cochrane library, and the Science Citation Index for published trials as well as ClinicalTrials.gov, and WHO ICTRP trial registers for ongoing or unreported studies. The search strategies, which combine the Cochrane sensitivity maximising RCT filter [49] with a combination of subject headings and free text terms relating to the interventions and diseases of interest, are provided in Appendix 1. Searches was updated periodically until October 2019.

Other resources

We will also search the references of all identified studies for additional studies eligible for inclusion. We will also contact the American Society of Peritoneal Surface Malignancies, the Canadian HIPEC

For peer review only

Collaborative Group (CHICG), The Peritoneal Surface Oncology Group International (PSOGI), and the study authors who agree to participate in this project for further studies.

Data collection and management

Selection of studies

Two review authors will independently screen the titles and abstracts of all records retrieved and make the final selection based on full text (after translation if required, i.e. there will be no language restrictions). We will document the process to enable completion of the PRISMA flow-chart. We will resolve discrepancies through discussion and arbitration.

Data collection

At the study level, we will record the contact details of the study author and the study contact, information required to assess the risk of bias, details of the treatment centres (name and the average number of CRS+HIPEC performed per year). At the participant level, we will collect the following details:

1. Centre at which treated
2. Patient demographics: age, gender, comorbidities, performance index
3. Cancer details (including severity)
4. Intervention details
5. Control details
6. Follow-up details
7. Outcome data
8. Resource utilisation data
    a. Operating time
    b. Quantity of blood and blood products transfused
    c. Length of hospital stay (including readmissions)

For peer review only

d. Length of intensive care unit stay

e. Chemotherapy regimen used in HIPEC and in control group if applicable

f. Proportion in whom surgery was performed and the nature of surgery in the control group

g. Additional surgery and other palliative treatments

These data will be sought for all patients randomised into each trial. Up to date follow-up will be requested in order to report on longer-term outcomes: the existing ethical approval for the studies usually cover collection of data.

The proposed data format and coding conventions for these data will be developed as part of the project to obtain the EVERPET-IPD data dictionary. Transfer guide will be developed as part of the project. Although the aim of the conventions is to facilitate data transfer, they are not essential. Data will be accepted in the format most convenient for the individual trial investigator or data centre, however, all personal identifiers (e.g. names) are to be removed before sharing. Data should be transferred by encrypted email or source ftp site. Further details are included in the data transfer guide.

Data checking and management

Once trial investigators have agreed to provide the IPD, they will be asked to sign a data transfer agreement that covers the transfer, use and storage of that data. By signing up to the agreement, investigators also declare that they have complied with all laws and regulations relating to the conduct of their studies and the collection of data as part of those studies.

On receipt, data will be cleaned and checked for accuracy, consistency and validity. This will include checks for missing data, randomisation integrity, follow-up and censoring. We will query any anomalies with the study contact to ensure that the data are represented accurately, and send a summary of the final dataset from each trial to the study contacts for verification.

For peer review only

Once checked and verified, we will store the trial data securely. Access to the data will be restricted to the Project Management Group, who are all trained in data protection and personal data confidentiality and who will act as custodians of the data under the terms of the data transfer agreement, which will be developed as part of this project. In line with that agreement, data will be deposited in the EVidencE Review of PEritoneal Tumours (EVERPET)-IPD repository.

Assessment of risk of bias in included studies

We will use the Cochrane risk of bias tool version 2 to assess the risk of bias in RCTs [50].

Meta-analysis of clinical effectiveness

Measures of treatment effect and data synthesis

We will use risk ratio for binary outcomes (proportion of people with serious and non-serious adverse events), mean difference (if same scales are reported in the studies) or standardised mean difference (if different scales are reported in the studies) for continuous outcomes (HRQoL), rate ratios for count outcomes (number of serious and non-serious adverse events), and hazard ratio for time-to-event outcomes (overall all-cause mortality and time to progression) with their respective 95% confidence intervals.

We will perform a two-step IPD, i.e. calculate the adjusted effect estimate from each included study and then perform a random-effects model meta-analysis using DerSimonian and Laird method [51] for binary outcomes and inverse variance method for other types of outcomes. The reason for choosing the two-step IPD over one-step IPD is the way the confounding factors are reported in the studies, for example, comorbidities can be reported as different types of performance indices and the extent of peritoneal disease can be reported in different ways [52, 53]. However, if we agree on an approximation to convert different performance indices into a single measure and convert the different measures of extent of peritoneal involvement into a single measure, we will perform a single-step meta-analysis to check the robustness of the two-step meta-analysis. We will test our assumptions in approximations (of the different performance indices into a single measure and

For peer review only

different measures of extent of peritoneal involvement into a single measure) by sensitivity analyses. We will use multilevel modelling to take the clustering of data in the studies into account for the one-step IPD meta-analysis, as the unit of analysis will be the individual participant.

Dealing with missing data

We will perform an intention-to-treat analysis whenever possible [54]. If data on the classification of the treatment as intervention or control is missing, and cannot be ascertained though discussion with trialists, we will exclude such participants. If outcome data are missing, we will use multiple imputation method if the data are likely to be missing at random or best-case and worst-case scenarios analysis if it is felt that the outcome data are not missing at random.

Assessment and investigation of heterogeneity

We will assess the clinical and methodological heterogeneity by carefully examining the characteristics and design of included trials. Clinical heterogeneity could be due to the type of participants included in the studies (performance index, stage of cancer, extent of peritoneal involvement, other organ involvement), different interventions (complete CRS or not, chemotherapy agents used), different controls (chemotherapy alone or CRS or both), whether complete CRS was achieved (if the control group was CRS), or different follow-up methods (routine imaging versus clinical examination). Different study designs and risk of bias may contribute to methodological heterogeneity. We will calculate and report the between-trial standard deviation and I^2 as measures of heterogeneity.

If we identify substantial clinical, methodological, or statistical heterogeneity, we will explore and address it in subgroup analyses and/or metaregression using participant level covariates on the sources of clinical heterogeneity mentioned above except for routine imaging which will be a trial-level covariate. All sources of methodological heterogeneity will be trial-level covariates.

Sensitivity analysis

We will perform the following sensitivity analyses to assess the impact of:

For peer review only

- data not missing at random
- non-participation in the IPD
- methods (two-step versus single-step) and model (fixed-effect versus random-effects model) used for meta-analysis
- using 'time from diagnosis' rather than 'time from randomisation' for defining 'time to disease progression'
- risk of bias.

Network meta-analysis

We will also perform a network meta-analysis of aggregate data to compare the three treatments:

1. CRS + HIPEC + systemic chemotherapy
2. CRS alone + systemic chemotherapy
3. Systemic chemotherapy

Reporting bias

We will assess reporting bias by the completeness of search.

Confidence in results

The uncertainty in results will be evaluated using the GRADE methodology [55].

Cost-effectiveness analysis

We will follow the NICE methodological standards for conducting our cost-effectiveness analysis [56].

Model

We will perform a model-based cost-utility analysis estimating mean costs and quality-adjusted life years (QALYs) per patient. We will compare CRS+HIPEC versus SoC in each of the three cancers by three separate cost-effectiveness analyses. The time horizon will be life-time time horizon. We will calculate the costs from the NHS and personal social services (PSS) perspective. We will discount the costs and utilities at the rate of 3.5% per annum [56].

For peer review only

We will create a decision tree model (one for each cancer) along the lines of the model that we used to compare two types of surgeries in pancreatic cancer [57]. Briefly, a patient with peritoneal metastases from one of the three cancers (colorectal cancer, ovarian cancer, or gastric cancer) and eligible for CRS+HIPEC can either undergo CRS+HIPEC or SoC. A proportion of patients undergoing CRS+HIPEC will have complete CRS (i.e. all macroscopic tumour is removed). A proportion of patients in whom CRS+HIPEC will develop complications (whether complete CRS was achieved or not), a proportion of whom may die within 90 days. Those who are alive at 90 days may die subsequently (a Markov model will be used to model this). The decision tree pathways in the people who had SoC will be identical: some will have complete CRS, some will have complications, some will die within 90 days, and some will die after 90 days.

Most of the information required for populating the decision tree (including resource utilisation data) will be obtained from the systematic review and IPD meta-analysis. For information not available from the systematic review and IPD meta-analysis, we will perform literature searches of NHS Economic Evaluation Database (NHS EED), the Health Economic Evaluations Database (HEED), MEDLINE, and EMBASE (for MEDLINE and EMBASE, we will combine the search strategy from Appendix 1 with sensitivity maximising 'economics' filter developed as a part of The Hedges Project of the Health Information Research Unit of McMaster University). We will also review the Cost-Effectiveness Analysis Registry (CEA) at Tufts University for information on quality of life. Currently, there is no HRG (Healthcare Resource Group) code available for CRS+HIPEC and SoC (which will vary according to the nature of the treatment). We will obtain resource utilisation data as part of the systematic review and IPD (please see above) and convert these to costs on the basis of NHS National Tariff, NHS National Schedule of Reference costs, British National Formulary, and/or local estimates as required.

We will assume that the people who die in each period will do so at a constant rate during the period and check whether this assumption is true using the IPD. If this assumption is not true, then we will use more complex models to mirror the survival curves based on the IPD. When no data

For peer review only

are available from the IPD or published sources, a range of values will be used in the model. We will tabulate the inputs used in the decision tree model and the source of these inputs in the project report.

Measuring cost-effectiveness

We will measure cost-effectiveness using net monetary benefits (NMBs). For each treatment, we will calculate the NMB as the mean QALYs per patient accruing to that treatment multiplied by decision-makers' maximum willingness to pay for a QALY (also referred to as the cost-effectiveness threshold), minus the mean cost per patient for the treatment. In the UK, the upper limit of the maximum willingness to pay for a QALY is £20,000 to £30,000 [56]. NMBs will be calculated using the base case parameter values to obtain the deterministic results, which do not depend on chance. The option with the highest NMB represents best value for money. The NMB for CRS+HIPEC minus the NMB for SoC is the incremental NMB. If the incremental NMB is positive then CRS+HIPEC represents better value for money; if it is negative, the SoC represents better value for money.

A probabilistic sensitivity analysis (PSA) will also be undertaken [56]. The PSA involves Monte Carlo simulation and takes variability of all selected inputs into account simultaneously. Distributions will be assigned to parameters to reflect the uncertainty with each parameter value. A random value from the corresponding distribution for each parameter will be selected (by the computer). This will generate an estimate of the mean cost and mean QALYs and the NMB associated with each treatment. This will be repeated 10,000 times and the results for each simulation will be noted. The mean costs, QALYs and NMB for each treatment will be calculated from the 10,000 simulations; these are probabilistic results because they depend on chance. We will increase the simulations as required by a stability test, which involves ensuring that the standard error when the PSA is repeated 30 times is within 2% of each other. The NMB will also calculated for each of the 10,000 simulations and the proportion of times each treatment had the highest NMB will be calculated for a range of values for the maximum willingness to pay for a QALY. These will be summarised graphically using cost-effectiveness acceptability curves. We will derive the 95% confidence intervals around the base

For peer review only

case values using the 2.5 and 97.5 percentiles calculated from the PSA. We will also perform a value of information analysis and calculate the expected value of perfect information and the expected value of partially perfect information.

For the deterministic univariate sensitivity analysis, each variable in the cost-effectiveness model will be varied one at a time. The results of the sensitivity analysis will be represented in the tornado diagram which reflects the variation in the NMB within the range of the lowest and highest value used for a parameter with all else equal. If the variation in the NMB includes 0, then there is uncertainty in the cost-effectiveness due to the variation of the parameter.

We will also perform various subgroup analyses guided by the results of the systematic reviews and IPD meta-analyses, but will include subgroup analysis of different types of control (i.e. CRS alone or systemic chemotherapy alone or both) as a minimum. We will also perform a sensitivity analysis using information from 'real-life' prospective data from Christie NHS foundation trust (and from other NHS specialist centres if such information is available).

We will follow the 'Consolidated Health Economic Evaluation Reporting Standards' (CHEERS) reporting checklist for reporting the cost-effectiveness analysis [58].

Patient and public involvement

A patient representative was involved in the preparation of this grant proposal and found that this research proposal was important to patients. Additional patient representatives will be identified. A patient representative will also be part of the research oversight committee.

For peer review only

Abbreviations

CEA	Cost-Effectiveness Analysis Registry
CHEERS	Consolidated Health Economic Evaluation Reporting Standards
CHICG	The Canadian HIPEC Collaborative Group
CRS	Cytoreductive surgery
EVERPET	EVidencE Review of PEritoneal Tumours
HEED	Health Economic Evaluations Database
HIPEC	Hyperthermic intraoperative peritoneal chemotherapy
HRG	Healthcare Resource Group
HRQoL	Health-related quality of life
HTA	Health technology assessments
IPD	Individual participant data
NHS	National Health Service
NHS EED	NHS Economic Evaluation Database
NMBs	Net monetary benefits
PMP	Pseudomyxoma peritonei
PRISMA-P	Preferred Reporting Items for Systematic Review and Meta-Analysis Protocols
PSA	Probabilistic sensitivity analysis
PSOGI	The Peritoneal Surface Oncology Group International
PSS	Personal social services
QALYs	Quality-adjusted life years
R0 resection	Resected completely
RCTs	Randomised controlled trials
RECIST	Response Evaluation Criteria in Solid Tumors
ROBIS	Risk of bias in systematic reviews
SoC	Standard of care

For peer review only

Declarations

PROSPERO registration

CRD42019130504

Ethics approval

This project was approved by the UCL Research Ethics Committee (Ethics number: 16023/001).

Consent to participate

Not applicable as this is a systematic review.

Consent for publication

All authors approved this manuscript for publication. There is no other participant information included.

Dissemination and reporting plan

The authorship of the systematic review manuscript will comprise the Project Management Group (the authors of this manuscript), International Advisory Group, representatives from the included trials and patient representatives. We aim to present the findings at appropriate international meetings and publish the review, irrespective of the findings, in a peer-reviewed journal.

Availability of data and materials

Summary data will be shared. Reidentification risk of anonymous data will be assessed at the end of the project and ethical approval will be sought for onward sharing of anonymous data, if the reidentification risk is considered negligible.

Competing interests/Disclosures

The clinical practice of the clinicians in the project: Mr Tim Mould, Mr Muntzer Mughal, Dr Mark Saunders, Mr Omer Aziz, and Prof Sarah O'Dwyer may be altered by the findings of the review.

For peer review only

Funding

This study is funded by the National Institute for Health Research (NIHR) HTA programme (HTA - Project: 17/135/02).

Role of funder: The funder sought independent peer review before funding and approved the protocol. All dissemination must be approved by the funder before dissemination.

Disclaimer: The views expressed are those of the author(s) and not necessarily those of the NIHR or the Department of Health and Social Care.

For peer review only

Sponsor

University College London

Authors contributions

Kurinchi Gurusamy and Claire Vale wrote the manuscript. The manuscript was critically revised by Elena Pizzo, R Bhanot, Brian Davidson, Tim Mould, Muntzer Mughal, Mark Saunders, Omer Aziz, Sarah O'Dwyer. Kurinchi Gurusamy is the guarantor of this manuscript.

Acknowledgements

We used the PRISMA-P checklist when writing our report ('Moher D, Shamseer L, Clarke M, Ghersi D, Liberati A, Petticrew M, Shekelle P, Stewart LA. Preferred Reporting Items for Systematic Review and Meta-Analysis Protocols (PRISMA-P) 2015 statement. Syst Rev. 2015;4(1):1').

Licence

I, the Submitting Author has the right to grant and does grant on behalf of all authors of the Work (as defined in the below author licence), an exclusive licence on a worldwide, perpetual, irrevocable, royalty-free basis to BMJ Publishing Group Ltd ("BMJ") its licensees and where the relevant Journal is co-owned by BMJ to the co-owners of the Journal, to publish the Work in BMJ Open and any other BMJ products and to exploit all rights, as set out in our licence. The Submitting Author accepts and understands that any supply made under these terms is made by BMJ to the Submitting Author.

References

1. International Agency for Research on Cancer (World Health Organization): **Estimated number of incident cases, both sexes, all cancers excluding non-melanoma skin cancer, worldwide in 2012**. https://gco.iarc.fr/today/online-analysis-table?mode=cancer&mode_population=continents&population=900&sex=0&cancer=29&type=0&statistic=0&prevalence=0&color_palette=default *(accessed on 3rd December 2017)* 2017.

2. Lemmens VE, Klaver YL, Verwaal VJ, Rutten HJ, Coebergh JWW, de Hingh IH: **Predictors and survival of synchronous peritoneal carcinomatosis of colorectal origin: A population-based study**. *International Journal of Cancer* 2011, **128**(11):2717-2725.

3. Riihimäki M, Hemminki A, Sundquist J, Hemminki K: **Patterns of metastasis in colon and rectal cancer**. *Scientific Reports* 2016, **6**:29765.

4. Segelman J, Granath F, Holm T, Machado M, Mahteme H, Martling A: **Incidence, prevalence and risk factors for peritoneal carcinomatosis from colorectal cancer**. *British Journal of Surgery* 2012, **99**(5):699-705.

5. van Gestel YRBM, Thomassen I, Lemmens VEPP, Pruijt JFM, van Herk-Sukel MPP, Rutten HJT, Creemers GJ, de Hingh IHJT: **Metachronous peritoneal carcinomatosis after curative treatment of colorectal cancer**. *European Journal of Surgical Oncology (EJSO)* 2014, **40**(8):963-969.

6. Baratti D, Kusamura S, Pietrantonio F, Guaglio M, Niger M, Deraco M: **Progress in treatments for colorectal cancer peritoneal metastases during the years 2010-2015. A systematic review**. *Critical Reviews in Oncology/Hematology* 2016, **100**:209-222.

7. Thomassen I, van Gestel YR, van Ramshorst B, Luyer MD, Bosscha K, Nienhuijs SW, Lemmens VE, de Hingh IH: **Peritoneal carcinomatosis of gastric origin: A population-based study on incidence, survival and risk factors**. *International Journal of Cancer* 2014, **134**(3):622-628.

8. Quere P, Facy O, Manfredi S, Jooste V, Faivre J, Lepage C, Bouvier AM: **Epidemiology, Management, and Survival of Peritoneal Carcinomatosis from Colorectal Cancer: A Population-Based Study**. *Dis Colon Rectum* 2015, **58**(8):743-752.

For peer review only

9. Klaver YL, Lemmens VE, Creemers GJ, Rutten HJ, Nienhuijs SW, de Hingh IH: **Population-based survival of patients with peritoneal carcinomatosis from colorectal origin in the era of increasing use of palliative chemotherapy**. *Ann Oncol* 2011, **22**(10):2250-2256.

10. Hwang M, Jayakrishnan TT, Green DE, George B, Thomas JP, Groeschl RT, Erickson B, Pappas SG, Gamblin TC, Turaga KK: **Systematic review of outcomes of patients undergoing resection for colorectal liver metastases in the setting of extra hepatic disease**. *Eur J Cancer* 2014, **50**(10):1747-1757.

11. Spiliotis J, Halkia E, Lianos E, Kalantzi N, Grivas A, Efstathiou E, Giassas S: **Cytoreductive surgery and HIPEC in recurrent epithelial ovarian cancer: a prospective randomized phase III study**. *Annals of surgical oncology* 2015, **22**(5):1570-1575.

12. Yang XJ, Huang CQ, Suo T, Mei LJ, Yang GL, Cheng FL, Zhou YF, Xiong B, Yonemura Y, Li Y: **Cytoreductive surgery and hyperthermic intraperitoneal chemotherapy improves survival of patients with peritoneal carcinomatosis from gastric cancer: Final results of a phase III randomized clinical trial**. *Annals of surgical oncology* 2011, **18**(6):1575-1581.

13. Verwaal VJ, Van Ruth S, De Bree E, Van Slooten GW, Van Tinteren H, Boot H, Zoetmulder FAN: **Randomized trial of cytoreduction and hyperthermic intraperitoneal chemotherapy versus systemic chemotherapy and palliative surgery in patients with peritoneal carcinomatosis of colorectal cancer**. *Journal of Clinical Oncology* 2003, **21**(20):3737-3743.

14. Razenberg LG, van Gestel YR, Creemers GJ, Verwaal VJ, Lemmens VE, de Hingh IH: **Trends in cytoreductive surgery and hyperthermic intraperitoneal chemotherapy for the treatment of synchronous peritoneal carcinomatosis of colorectal origin in the Netherlands**. *Eur J Surg Oncol* 2015, **41**(4):466-471.

15. Verwaal VJ, Bruin S, Boot H, van Slooten G, van Tinteren H: **8-year follow-up of randomized trial: cytoreduction and hyperthermic intraperitoneal chemotherapy versus systemic chemotherapy in patients with peritoneal carcinomatosis of colorectal cancer**. *Annals of surgical oncology* 2008, **15**(9):2426-2432.

For peer review only

16. Elias D, Goere D, Dumont F, Honore C, Dartigues P, Stoclin A, Malka D, Boige V, Ducreux M: **Role of hyperthermic intraoperative peritoneal chemotherapy in the management of peritoneal metastases**. *Eur J Cancer* 2014, **50**(2):332-340.

17. Desiderio J, Chao J, Melstrom L, Warner S, Tozzi F, Fong Y, Parisi A, Woo Y: **The 30-year experience-A meta-analysis of randomised and high-quality non-randomised studies of hyperthermic intraperitoneal chemotherapy in the treatment of gastric cancer**. *European Journal of Cancer* 2017, **79**:1-14.

18. Schaaf L, van der Kuip H, Zopf W, Winter S, Munch M, Murdter TE, Thon KP, Steurer W, Aulitzky WE, Ulmer C: **A Temperature of 40 degrees C Appears to be a Critical Threshold for Potentiating Cytotoxic Chemotherapy In Vitro and in Peritoneal Carcinomatosis Patients Undergoing HIPEC**. *Ann Surg Oncol* 2015, **22**:S758-S765.

19. Seretis C, Youssef H: **Quality of life after cytoreductive surgery and intraoperative hyperthermic intraperitoneal chemotherapy for peritoneal surface malignancies: a systematic review**. *Eur J Surg Oncol* 2014, **40**(12):1605-1613.

20. Huang CQ, Min Y, Wang SY, Yang XJ, Liu Y, Xiong B, Yonemura Y, Li Y: **Cytoreductive surgery plus hyperthermic intraperitoneal chemotherapy improves survival for peritoneal carcinomatosis from colorectal cancer: A systematic review and meta-analysis of current evidence**. *Oncotarget* 2017, **8**(33):55657-55683.

21. Cavaliere D, Cirocchi R, Coccolini F, Fagotti A, Fambrini M, Federici O, Lorusso D, Vaira M, Ceresoli M, Delrio P *et al*: **1st Evidence-based Italian consensus conference on cytoreductive surgery and hyperthermic intraperitoneal chemotherapy for peritoneal carcinosis from ovarian cancer**. *Tumori* 2017, **103**(6):525-536.

22. Baratti D, Scivales A, Balestra MR, Ponzi P, Di Stasi F, Kusamura S, Laterza B, Deraco M: **Cost analysis of the combined procedure of cytoreductive surgery and hyperthermic intraperitoneal chemotherapy (HIPEC)**. *Ejso* 2010, **36**(5):463-469.

For peer review only

23. Bonastre J, Chevalier J, Elias D, Classe JM, Ferron G, Guilloit JM, Marchal F, Meeus P, De Pouvourville G: **Cost-effectiveness of intraperitoneal chemohyperthermia in the treatment of peritoneal carcinomatosis from colorectal cancer**. *Value in Health* 2008, **11**(3):347-353.

24. Chua TC, Martin S, Saxena A, Liauw W, Yan TD, Zhao J, Lok I, Morris DL: **Evaluation of the cost-effectiveness of cytoreductive surgery and hyperthermic intraperitoneal chemotherapy (peritonectomy) at the st george hospital peritoneal surface malignancy program**. *Annals of Surgery* 2010, **251**(2):323-329.

25. Lee Z, Chia C, Teo M: **Is cytoreductive surgery and hyperthermic intraperitoneal chemotherapy cost-effective for metastatic colorectal cancer?** *Annals of surgical oncology* 2016, **23**(1 Suppl):S77.

26. Ludwigs K, Breimer ME, Brorson F, Carlsson G, Daxberg EL, Hjalmarsson Y, Skullman S, Strandell A, Jivegård L: **Cytoreductive surgery and intraperitoneal chemotherapy (HIPEC or EPIC) in patients with colorectal adenocarcinoma and peritoneal carcinomatosis. (Structured abstract)**. *Health Technology Assessment Database* 2013(4).

27. Naffouje SA, O'Donoghue C, Salti GI: **Evaluation of cytoreductive surgery and hyperthermic intraperitoneal chemotherapy in a community setting: A cost-utility analysis of a hospital's initial experience and reflections on the health care system**. *Journal of Surgical Oncology* 2016, **113**(5):544-547.

28. Tentes AAK, Pallas N, Korakianitis O, Mavroudis C, Spiridonidou A, Zorbas G, Popidis S, Papadoniou N, Darladima V, Smyrnis A *et al*: **The cost of cytoreductive surgery and perioperative intraperitoneal chemotherapy in the treatment of peritoneal malignancy in one Greek institute**. *Journal of B.U.ON.* 2012, **17**(4):776-780.

29. Cao C, Yan TD, Black D, Morris DL: **A systematic review and meta-analysis of cytoreductive surgery with perioperative intraperitoneal chemotherapy for peritoneal carcinomatosis of colorectal origin**. *Annals of surgical oncology* 2009, **16**(8):2152-2165.

For peer review only

30. Ceelen WP, Hesse U, de Hemptinne B, Pattyn P: **Hyperthermic intraperitoneal chemoperfusion in the treatment of locally advanced intra-abdominal cancer**. *British Journal of Surgery* 2000, **87**(8):1006-1015.

31. Chia CS, Seshadri RA, Kepenekian V, Vaudoyer D, Passot G, Glehen O: **Survival outcomes after Cytoreductive surgery and hyperthermic intraperitoneal chemotherapy for peritoneal carcinomatosis from gastric cancer: A systematic review**. *Pleura and Peritoneum* 2016, **1**(2):67-77.

32. Chua TC, Esquivel J, Pelz JO, Morris DL: **Summary of current therapeutic options for peritoneal metastases from colorectal cancer**. *Journal of Surgical Oncology* 2013, **107**(6):566-573.

33. Di Vita M, Cappellani A, Piccolo G, Zanghi A, Cavallaro A, Bertola G, Bolognese A, Facchini G, D'Aniello C, Di Francia R *et al*: **The role of HIPEC in the treatment of peritoneal carcinomatosis from gastric cancer: Between lights and shadows**. *Anti-Cancer Drugs* 2015, **26**(2):123-138.

34. Eveno C, Pocard M: **Randomized controlled trials evaluating Cytoreductive surgery (CRS) and hyperthermic intraperitoneal chemotherapy (HIPEC) in prevention and therapy of peritoneal metastasis: A Systematic review**. *Pleura and Peritoneum* 2016, **1**(4):169-182.

35. He TF, Chen ZR, Xing CG: **Cytoreductive surgery combined with intraperitoneal chemotherapy in the treatment of colorectal peritoneal metastasis: a meta-analysis**. *International Journal of Clinical and Experimental Medicine* 2016, **9**(11):20562-20570.

36. Hotouras A, Desai D, Bhan C, Murphy J, Lampe B, Sugarbaker PH: **Heated IntraPEritoneal Chemotherapy (HIPEC) for patients with recurrent ovarian cancer: A systematic literature review**. *International Journal of Gynecological Cancer* 2016, **26**(4):661-670.

37. Huo YR, Richards A, Liauw W, Morris DL: **Hyperthermic intraperitoneal chemotherapy (HIPEC) and cytoreductive surgery (CRS) in ovarian cancer: A systematic review and meta-analysis**. *European Journal of Surgical Oncology* 2015, **41**(12):1578-1589.

38. Lopez-Lopez V, Cascales-Campos PA, Schneider MA, Gil J, Gil E, Gomez-Hidalgo NR, Parrilla P: **Cytoreductive surgery and hyperthermic intraperitoneal chemotherapy (HIPEC) in elderly patients. A systematic literature review**. *Surgical Oncology-Oxford* 2016, **25**(4):378-384.

For peer review only

39. Mirnezami R, Moran BJ, Harvey K, Cecil T, Chandrakumaran K, Carr N, Mohamed F, Mirnezami AH: **Cytoreductive surgery and intraperitoneal chemotherapy for colorectal peritoneal metastases**. *World Journal of Gastroenterology* 2014, **20**(38):14018-14032.

40. Kireeva GS, Gafton GI, Guseynov KD, Senchik KY, Belyaeva OA, Bespalov VG, Panchenko AV, Maydin MA, Belyaev AM: **HIPEC in patients with primary advanced ovarian cancer: Is there a role? A systematic review of short- and long-term outcomes**. *Surg Oncol* 2018, **27**(2):251-258.

41. Morano WF, Khalili M, Chi DS, Bowne WB, Esquivel J: **Clinical studies in CRS and HIPEC: Trials, tribulations, and future directions-A systematic review**. *J Surg Oncol* 2018, **117**(2):245-259.

42. Whiting P, Savovic J, Higgins JP, Caldwell DM, Reeves BC, Shea B, Davies P, Kleijnen J, Churchill R, group R: **ROBIS: A new tool to assess risk of bias in systematic reviews was developed**. *J Clin Epidemiol* 2016, **69**:225-234.

43. Ubago-Pérez R, Matas-Hoces A, Beltrán-Calvo C, Romero-Tabares A: **Hyperthermic intraperitoneal chemotherapy. Efficacy and safety in the treatment of ovarian cancer peritoneal carcino matosis (Structured abstract)**. *Health Technology Assessment Database* 2013(4).

44. Riley RD, Lambert PC, Abo-Zaid G: **Meta-analysis of individual participant data: rationale, conduct, and reporting**. *BMJ* 2010, **340**.

45. Tierney JF, Vale C, Riley R, Smith CT, Stewart L, Clarke M, Rovers M: **Individual Participant Data (IPD) Meta-analyses of Randomised Controlled Trials: Guidance on Their Use**. *PLoS Med* 2015, **12**(7):e1001855.

46. Clavien PA, Barkun J, de Oliveira ML, Vauthey JN, Dindo D, Schulick RD, de Santibanes E, Pekolj J, Slankamenac K, Bassi C *et al*: **The Clavien-Dindo classification of surgical complications: five-year experience**. *Ann Surg* 2009, **250**(2):187-196.

47. Dindo D, Demartines N, Clavien PA: **Classification of surgical complications: a new proposal with evaluation in a cohort of 6336 patients and results of a survey**. *Ann Surg* 2004, **240**(2):205-213.

48. Therasse P, Arbuck SG, Eisenhauer EA, Wanders J, Kaplan RS, Rubinstein L, Verweij J, Van Glabbeke M, van Oosterom AT, Christian MC *et al*: **New guidelines to evaluate the response to**

For peer review only

treatment in solid tumors. European Organization for Research and Treatment of Cancer, National Cancer Institute of the United States, National Cancer Institute of Canada. *J Natl Cancer Inst* 2000, **92**(3):205-216.

49. Higgins J, Green S, (editors): **Cochrane Handbook for Systematic Reviews of Interventions Version 5.1.0 [updated March 2011]**. *The Cochrane Collaboration. Available from www.handbook.cochrane.org* 2011.

50. Sterne JAC, Savović J, Page MJ, Elbers RG, Blencowe NS, Boutron I, Cates CJ, Cheng H-Y, Corbett MS, Eldridge SM *et al*: **RoB 2: a revised tool for assessing risk of bias in randomised trials**. *BMJ* 2019, **366**:l4898.

51. DerSimonian R, Laird N: **Meta-analysis in clinical trials**. *Control Clin Trials* 1986, **7**(3):177-188.

52. Ng JL, Ong WS, Chia CS, Tan GH, Soo KC, Teo MC: **Prognostic Relevance of the Peritoneal Surface Disease Severity Score Compared to the Peritoneal Cancer Index for Colorectal Peritoneal Carcinomatosis**. *Int J Surg Oncol* 2016, **2016**:2495131.

53. Esquivel J, Lowy AM, Markman M, Chua T, Pelz J, Baratti D, Baumgartner JM, Berri R, Bretcha-Boix P, Deraco M *et al*: **The American Society of Peritoneal Surface Malignancies (ASPSM) Multiinstitution Evaluation of the Peritoneal Surface Disease Severity Score (PSDSS) in 1,013 Patients with Colorectal Cancer with Peritoneal Carcinomatosis**. *Annals of surgical oncology* 2014, **21**(13):4195-4201.

54. Newell DJ: **Intention-to-treat analysis: implications for quantitative and qualitative research**. *International journal of epidemiology* 1992, **21**(5):837-841.

55. Guyatt G, Oxman AD, Akl EA, Kunz R, Vist G, Brozek J, Norris S, Falck-Ytter Y, Glasziou P, DeBeer H *et al*: **GRADE guidelines: 1. Introduction-GRADE evidence profiles and summary of findings tables**. *J Clin Epidemiol* 2011, **64**(4):383-394.

56. National Institute for Health and Care Excellence: **Guide to the methods of technology appraisal 2013**. *https://www.nice.org.uk/process/pmg9/chapter/foreword (accessed on 4th June 2018)* 2013.

For peer review only

57. Gurusamy KS, Riviere D, van Laarhoven CJH, Besselink M, Abu-Hilal M, Davidson BR, Morris S: **Cost-effectiveness of laparoscopic versus open distal pancreatectomy for pancreatic cancer**. *PLoS One* 2017, **12**(12):e0189631.

58. Husereau D, Drummond M, Petrou S, Carswell C, Moher D, Greenberg D, Augustovski F, Briggs AH, Mauskopf J, Loder E *et al*: **Consolidated Health Economic Evaluation Reporting Standards (CHEERS)--explanation and elaboration: a report of the ISPOR Health Economic Evaluation Publication Guidelines Good Reporting Practices Task Force**. *Value Health* 2013, **16**(2):231-250.

For peer review only

Appendix 1: Search Strategies

Medline

1. Hyperthermia, Induced/

2. ((hyperthermic or heated) adj3 (intraperitoneal or intra-peritoneal) adj3 (chemotherapy or chemotherapies)).ti,ab.

3. (intraperitoneal adj3 chemohyperthermia).ti,ab.

4. (HIPEC or IPHC or HIIC).ti,ab.

5. 1 or 2 or 3 or 4

6. Cytoreduction Surgical Procedures/

7. ((cytoreductive or cytoreduction or debulking) adj3 (surgery or surgeries or surgical or procedure or procedures)).ti,ab.

8. 6 or 7

9. 5 or 8

10. exp Colorectal Neoplasms/

11. exp Ovarian Neoplasms/

12. Stomach Neoplasms/

13. ((colorectal or bowel or colon or colonic or rectum or rectal or ovary or ovaries or ovarian or gastric or stomach) adj3 (cancer or cancers or carcinoma or carcinomas or tumour or tumours or tumor or tumors or neoplasm or neoplasms)).ti,ab.

14. 10 or 11 or 12 or 13

15. 9 and 14

16. randomized controlled trial.pt.

17. controlled clinical trial.pt.

18. randomized.ab.

19. placebo.ab.

For peer review only

20. drug therapy.fs.

21. randomly.ab.

22. trial.ab.

23. groups.ab.

24. 16 or 17 or 18 or 19 or 20 or 21 or 22 or 23

25. exp animals/ not humans.sh.

26. 24 not 25

27. 15 and 26

28. (cost: or cost benefit analys: or health care costs).mp.

29. 15 and 28

30. 27 or 29

Embase

1. hyperthermic intraperitoneal chemotherapy/

2. ((hyperthermic or heated) adj3 (intraperitoneal or intra-peritoneal) adj3 (chemotherapy or chemotherapies)).ti,ab.

3. (intraperitoneal adj3 chemohyperthermia).ti,ab.

4. (HIPEC or IPHC or HIIC).ti,ab.

5. 1 or 2 or 3 or 4

6. cytoreductive surgery/

7. ((cytoreductive or cytoreduction or debulking) adj3 (surgery or surgeries or surgical or procedure or procedures)).ti,ab.

8. 6 or 7

9. 5 or 8

10. exp colon cancer/

11. exp rectum cancer/

12. exp ovary cancer/

For peer review only

13. exp stomach cancer/

14. ((colorectal or bowel or colon or colonic or rectum or rectal or ovary or ovaries or ovarian or gastric or stomach) adj3 (cancer or cancers or carcinoma or carcinomas or tumour or tumours or tumor or tumors or neoplasm or neoplasms)).ti,ab.

15. 10 or 11 or 12 or 13 or 14

16. 9 and 15

17. exp crossover-procedure/ or exp double-blind procedure/ or exp randomized controlled trial/ or single-blind procedure/

18. (((((random* or factorial* or crossover* or cross over* or cross-over* or placebo* or double*) adj blind*) or single*) adj blind*) or assign* or allocat* or volunteer*).af.

19. 17 or 18

20. 16 and 19

21. (cost or costs).tw.

22. 16 and 21

23. 20 or 22

Cochrane

#1 MeSH descriptor: [Hyperthermia, Induced] this term only

#2 ((hyperthermic or heated) near/3 (intraperitoneal or intra-peritoneal) near/3 (chemotherapy or chemotherapies))

#3 (intraperitoneal near/3 chemohyperthermia)

#4 (HIPEC or IPHC or HIIC)

#5 #1 or #2 or #3 or #4

#6 MeSH descriptor: [Cytoreduction Surgical Procedures] this term only

#7 ((cytoreductive or cytoreduction or debulking) near/3 (surgery or surgeries or surgical or procedure or procedures))

#8 #6 or #7

For peer review only

#9 #5 or #8

#10 MeSH descriptor: [Colorectal Neoplasms] explode all trees

#11 MeSH descriptor: [Ovarian Neoplasms] explode all trees

#12 MeSH descriptor: [Stomach Neoplasms] this term only

#13 ((colorectal or bowel or colon or colonic or rectum or rectal or ovary or ovaries or ovarian or gastric or stomach) near/3 (cancer or cancers or carcinoma or carcinomas or tumour or tumours or tumor or tumors or neoplasm or neoplasms))

#14 #10 or #11 or #12 or #13

#15 #9 and #14

Science Citation Index

1 TS=((hyperthermic or heated) near/3 (intraperitoneal or intra-peritoneal) near/3 (chemotherapy or chemotherapies))

2 TS=(intraperitoneal near/3 chemohyperthermia)

3 TS=(HIPEC or IPHC or HIIC)

4 #3 OR #2 OR #1

5 TS=((cytoreductive or cytoreduction or debulking) near/3 (surgery or surger-ies or surgical or procedure or procedures))

6 #5 or #4

7 TS=((colorectal or bowel or colon or colonic or rectum or rectal or ovary or ovaries or ovarian or gastric or stomach) near/3 (cancer or cancers or carci-noma or carcinomas or tumour or tumours or tumor or tumors or neoplasm or neoplasms))

#8 TS=(random* or placebo* or blind* or meta-analysis or cost or costs)

#9 #8 AND #7 AND #6

For peer review only

WHO trials register

Condition: colorectal OR bowel OR colon OR colonic OR rectum OR rectal OR ovary OR ovaries OR ovarian OR gastric OR stomach

Intervention: HIPEC OR hyperthermic intraperitoneal chemotherapy OR IPHC OR intraperitoneal chemohyperthermia OR HIIC OR heated intraoperative intraperitoneal chemotherapy OR cytoreductive surgery OR CRS

ClinicalTrials.gov

Condition: colorectal OR bowel OR colon OR colonic OR rectum OR rectal OR ovary OR ovaries OR ovarian OR gastric OR stomach

Study Type: Interventional Studies (Clinical Trials)

Intervention/treatment: HIPEC OR hyperthermic intraperitoneal chemotherapy OR IPHC OR intraperitoneal chemohyperthermia OR HIIC OR heated intraoperative intraperitoneal chemotherapy OR cytoreductive surgery OR CRS

Interventional studies, phase 2,3,4

Interventional Studies | colorectal OR bowel OR colon OR colonic OR rectum OR rectal OR ovary OR ovaries OR ovarian OR gastric OR stomach | HIPEC OR hyperthermic intraperitoneal chemotherapy OR IPHC OR intraperitoneal chemohyperthermia OR HIIC OR heated intraoperative intraperitoneal chemotherapy OR cytoreductive surgery OR CRS | Phase 2, 3, 4

Cost-Effectiveness Analysis (CEA) Registry

The following terms were searched:

Hyperthermic

Cytoreduction

Cytoreductive

For peer review only

Reporting checklist for protocol of a systematic review.

Based on the PRISMA-P guidelines.

Instructions to authors

Complete this checklist by entering the page numbers from your manuscript where readers will find each of the items listed below.

Your article may not currently address all the items on the checklist. Please modify your text to include the missing information. If you are certain that an item does not apply, please write "n/a" and provide a short explanation.

Upload your completed checklist as an extra file when you submit to a journal.

In your methods section, say that you used the PRISMA-Preporting guidelines, and cite them as:

Moher D, Shamseer L, Clarke M, Ghersi D, Liberati A, Petticrew M, Shekelle P, Stewart LA. Preferred Reporting Items for Systematic Review and Meta-Analysis Protocols (PRISMA-P) 2015 statement. Syst Rev. 2015;4(1):1.

		Reporting Item	Page Number
**Title**			
Identification	#1a	Identify the report as a protocol of a systematic review	1
Update	#1b	If the protocol is for an update of a previous systematic review, identify as such	NA

For peer review only

**Registration**			
	#2	If registered, provide the name of the registry (such as PROSPERO) and registration number	3
**Authors**			
Contact	#3a	Provide name, institutional affiliation, e-mail address of all protocol authors; provide physical mailing address of corresponding author	1-2
Contribution	#3b	Describe contributions of protocol authors and identify the guarantor of the review	3
**Amendments**			
	#4	If the protocol represents an amendment of a previously completed or published protocol, identify as such and list changes; otherwise, state plan for documenting important protocol amendments	NA
**Support**			
Sources	#5a	Indicate sources of financial or other support for the review	3
Sponsor	#5b	Provide name for the review funder and / or sponsor	3
Role of sponsor or funder	#5c	Describe roles of funder(s), sponsor(s), and / or institution(s), if any, in developing the protocol	3
**Introduction**			
Rationale	#6	Describe the rationale for the review in the context of what	6-8

For peer review only

		is already known	
Objectives	#7	Provide an explicit statement of the question(s) the review will address with reference to participants, interventions, comparators, and outcomes (PICO)	8-9
**Methods**			
Eligibility criteria	#8	Specify the study characteristics (such as PICO, study design, setting, time frame) and report characteristics (such as years considered, language, publication status) to be used as criteria for eligibility for the review	9-10
Information sources	#9	Describe all intended information sources (such as electronic databases, contact with study authors, trial registers or other grey literature sources) with planned dates of coverage	10-11
Search strategy	#10	Present draft of search strategy to be used for at least one electronic database, including planned limits, such that it could be repeated	Supplement 1-5
Study records - data management	#11a	Describe the mechanism(s) that will be used to manage records and data throughout the review	11-13
Study records - selection process	#11b	State the process that will be used for selecting studies (such as two independent reviewers) through each phase of the review (that is, screening, eligibility and inclusion in meta-analysis)	11
Study records -	#11c	Describe planned method of extracting data from reports	11-13

For peer review only

data collection process		(such as piloting forms, done independently, in duplicate), any processes for obtaining and confirming data from investigators	
Data items	#12	List and define all variables for which data will be sought (such as PICO items, funding sources), any pre-planned data assumptions and simplifications	11-13
Outcomes and prioritization	#13	List and define all outcomes for which data will be sought, including prioritization of main and additional outcomes, with rationale	10
Risk of bias in individual studies	#14	Describe anticipated methods for assessing risk of bias of individual studies, including whether this will be done at the outcome or study level, or both; state how this information will be used in data synthesis	13
Data synthesis	#15a	Describe criteria under which study data will be quantitatively synthesised	13-14
Data synthesis	#15b	If data are appropriate for quantitative synthesis, describe planned summary measures, methods of handling data and methods of combining data from studies, including any planned exploration of consistency (such as I2, Kendall's τ)	13-14
Data synthesis	#15c	Describe any proposed additional analyses (such as sensitivity or subgroup analyses, meta-regression)	14-15
Data synthesis	#15d	If quantitative synthesis is not appropriate, describe the type of summary planned	NA

For peer review only

Meta-bias(es)	#16	Specify any planned assessment of meta-bias(es) (such as publication bias across studies, selective reporting within studies)	15
Confidence in cumulative evidence	#17	Describe how the strength of the body of evidence will be assessed (such as GRADE)	NA

The PRISMA-P checklist is distributed under the terms of the Creative Commons Attribution License CC-BY 4.0. This checklist was completed on 10. April 2020 using https://www.goodreports.org/, a tool made by the EQUATOR Network in collaboration with Penelope.ai

For peer review only

BMJ Open

Cytoreductive surgery (CRS) with hyperthermic intraoperative peritoneal chemotherapy (HIPEC) versus standard of care (SoC) in people with peritoneal metastases from colorectal, ovarian or gastric origin: protocol for a systematic review and individual participant data (IPD) meta-analyses of effectiveness and cost-effectiveness

Journal:	*BMJ Open*
Manuscript ID	bmjopen-2020-039314.R1
Article Type:	Protocol
Date Submitted by the Author:	17-Apr-2020

Complete List of Authors:	Gurusamy, Kurinchi; University College London,<br>Vale, Claire; MRC Clinical Trials Unit at UCL, Meta-analysis Group<br>Pizzo, Elena; University College London, Applied Health Research<br>Bhanot, R; University College London, Patient representative (undisclosed)<br>Davidson, Brian; Royal Free London NHS Foundation Trust, HPB Surgery<br>Mould, Tim; University College London Hospitals NHS Foundation Trust<br>Mughal, Muntzer; University College London Hospitals NHS Foundation Trust,<br>Saunders, Mark ; The Christie NHS Foundation Trust,<br>Aziz, Omer; The Christie NHS Foundation Trust<br>O'Dwyer, Sarah; The Christie NHS Foundation Trust

**Primary Subject Heading**:	Oncology
Secondary Subject Heading:	Surgery, Health economics, Obstetrics and gynaecology
Keywords:	HEALTH ECONOMICS, CHEMOTHERAPY, QUALITATIVE RESEARCH, ONCOLOGY, SURGERY

SCHOLARONE™
Manuscripts

I, the Submitting Author has the right to grant and does grant on behalf of all authors of the Work (as defined in the below author licence), an exclusive licence and/or a non-exclusive licence for contributions from authors who are: i) UK Crown employees; ii) where BMJ has agreed a CC-BY licence shall apply, and/or iii) in accordance with the terms applicable for US Federal Government officers or employees acting as part of their official duties; on a worldwide, perpetual, irrevocable, royalty-free basis to BMJ Publishing Group Ltd ("BMJ") its licensees and where the relevant Journal is co-owned by BMJ to the co-owners of the Journal, to publish the Work in this journal and any other BMJ products and to exploit all rights, as set out in our licence.

The Submitting Author accepts and understands that any supply made under these terms is made by BMJ to the Submitting Author unless you are acting as an employee on behalf of your employer or a postgraduate student of an affiliated institution which is paying any applicable article publishing charge ("APC") for Open Access articles. Where the Submitting Author wishes to make the Work available on an Open Access basis (and intends to pay the relevant APC), the terms of reuse of such Open Access shall be governed by a Creative Commons licence – details of these licences and which Creative Commons licence will apply to this Work are set out in our licence referred to above.

Other than as permitted in any relevant BMJ Author's Self Archiving Policies, I confirm this Work has not been accepted for publication elsewhere, is not being considered for publication elsewhere and does not duplicate material already published. I confirm all authors consent to publication of this Work and authorise the granting of this licence.

For peer review only

Cytoreductive surgery (CRS) with hyperthermic intraoperative peritoneal chemotherapy (HIPEC) versus standard of care (SoC) in people with peritoneal metastases from colorectal, ovarian or gastric origin: protocol for a systematic review and individual participant data (IPD) meta-analyses of effectiveness and cost-effectiveness

Prof Kurinchi Gurusamy PhD[1], Dr Claire Vale PhD[2], Dr Elena Pizzo PhD[3], Mr R Bhanot BSc[1], Prof Brian Davidson MD FRCS[1], Mr Tim Mould DM FRCOG[4], Prof Muntzer Mughal FRCS[1], Dr Mark Saunders PhD[5], Mr Omer Aziz PhD[5], Prof Sarah O'Dwyer MD FRCS[5]

[1] Division of Surgery and Interventional Science, University College London, London

[2] MRC trials Unit, University College London

[3] Centre of Applied Health Research, University College London

[4] University College London NHS Foundation Trust

[5] The Christie NHS Foundation Trust

Corresponding author: Prof Kurinchi Gurusamy

Division of Surgery and Interventional Science, University College London

Hampstead Campus, 9th Floor, Royal Free Hospital

Rowland Hill Street

London

NW3 2PF

United Kingdom

Email: k.gurusamy@ucl.ac.uk

Tel: +44 (0)20 8016 8116

For peer review only

Contact details of authors

Kurinchi Gurusamy: Division of Surgery and Interventional Science, University College London, Hampstead Campus, Royal Free Hospital, Rowland Hill Street, London, NW3 2PF, United Kingdom; k.gurusamy@ucl.ac.uk

Claire Vale: MRC trials Unit, University College London, 90 High Holborn, London, WC1V 6LJ; claire.vale@ucl.ac.uk

Elena Pizzo: Centre of Applied Health Research, University College London, 1-19 Torrington Place, London WC1E 7HB; e.pizzo@ucl.ac.uk

R Bhanot: patient representative (first name and email to be disclosed on a need to know basis; address will not be disclosed)

Brian Davidson: Division of Surgery and Interventional Science, University College London, Hampstead Campus, Royal Free Hospital, Rowland Hill Street, London, NW3 2PF, United Kingdom, b.davidson@ucl.ac.uk

Tim Mould: University College London NHS Foundation Trust, 235 Euston Rd, Bloomsbury, London NW1 2BU; t.mould@nhs.net

Muntzer Mughal: Division of Surgery and Interventional Science, University College London, Bloomsbury Campus, Charles Bell House. 43-45 Foley Street, London, W1W 7TS; muntzer.mughal@nhs.net

Mark Saunders: The Christie NHS Foundation Trust, Christie Hospital, Palatine Rd, Withington, Manchester M20 3JJ; Mark.Saunders@christie.nhs.uk

Omer Aziz: Christie Hospital, Palatine Rd, Withington, Manchester M20 3JJ; Omer.Aziz@christie.nhs.uk

Sarah O'Dwyer: Christie Hospital, Palatine Rd, Withington, Manchester M20 3JJ; Sarah.O'Dwyer@christie.nhs.uk

For peer review only

Ethics approval: This project was approved by the UCL Research Ethics Committee (Ethics number: 16023/001).

PROSPERO registration: CRD42019130504

Funding: This study is funded by the National Institute for Health Research (NIHR) HTA programme (HTA - Project: 17/135/02).

Role of funder: The funder sought independent peer review before funding and approved the protocol. All dissemination must be approved by the funder before dissemination.

Disclaimer: The views expressed are those of the author(s) and not necessarily those of the NIHR or the Department of Health and Social Care.

Sponsor: University College London

Disclosures: The clinical practice of the clinicians in the project: Mr Tim Mould, Mr Muntzer Mughal, Dr Mark Saunders, Mr Omer Aziz, and Prof Sarah O'Dwyer may be altered by the findings of the review.

Author contributions: Kurinchi Gurusamy and Claire Vale wrote the manuscript. The manuscript was critically revised by Elena Pizzo, R Bhanot, Brian Davidson, Tim Mould, Muntzer Mughal, Mark Saunders, Omer Aziz, Sarah O'Dwyer. Kurinchi Gurusamy is the guarantor of this manuscript.

Keywords: systematic review, individual participant data, meta-analysis, peritoneal metastases, hyperthermic intraoperative peritoneal chemotherapy

Word count: 3725 words

For peer review only

Abstract

Introduction: There is uncertainty about whether cytoreductive surgery (CRS) + hyperthermic intraoperative peritoneal chemotherapy (HIPEC) improves survival and/or quality of life compared to standard of care (SoC) in people with peritoneal metastases who can withstand major surgery.

Primary objectives: To compare the relative benefits and harms of CRS+HIPEC versus SoC in people with peritoneal metastases from colorectal, ovarian, or gastric cancers eligible to undergo CRS+HIPEC by a systematic review and individual participant data (IPD) meta-analysis.

Secondary objectives: To compare the cost-effectiveness of CRS+HIPEC versus SoC from a National Health Service (NHS) and personal social services (PSS) perspective using a model-based cost-utility analysis.

Methods and analysis: We will perform a systematic review of literature by updating the searches from MEDLINE, EMBASE, Cochrane library, Science Citation Index as well as trial registers. Two members of our team will independently screen the search results and identify randomised controlled trials (RCTs) comparing CRS+HIPEC versus SoC for inclusion based on full texts for articles shortlisted during screening. We will assess the risk of bias in the trials and obtain data related to baseline prognostic characteristics, details of intervention and control, and outcome data related to overall survival, disease progression, health-related quality of life, treatment related complications, and resource utilisation data. Using individual participant data (IPD), we will perform a two-step IPD, i.e. calculate the adjusted effect estimate from each included study and then perform a random-effects model meta-analysis. We will perform various subgroup analyses, metaregression, and sensitivity analyses. We will also perform a model-based cost-utility analysis to assess whether CRS+HIPEC is cost-effective in the NHS setting.

Ethics and Dissemination: This project was approved by the UCL Research Ethics Committee (Ethics number: 16023/001). We aim to present the findings at appropriate international meetings and publish the review, irrespective of the findings, in a peer-reviewed journal.

For peer review only

Article summary

Strengths and limitations of this study

- This study utilises individual participant data, which has several advantages over collecting summary data.
- This study will identify the uncertainty of evidence and help in the identification of whether further research is necessary on the topic and how future research should be performed.
- Outcome data may not be available from the randomised controlled trials, even if they are important to patients and healthcare professionals.

For peer review only

Background and rationale

What is the problem being addressed?

Approximately 7 million people worldwide and 160,000 people in UK develop colorectal, ovarian, or gastric cancer each year [1], of whom 8% to 50% develop peritoneal metastases. The peritoneum is one of the commonest sites of metastases from these cancers [2-8], and is often the only site of metastases [7-9]. In general, people with peritoneal metastases have poorer prognosis than those with other metastases (liver or lung) [10], with median reported survival ranging from 6 to 24 months depending on from the primary cancers and treatment received [11-13].

Treatment of peritoneal metastases

The current standard of care of people with peritoneal metastases from these cancers is systemic chemotherapy alone or in combination with either cytoreductive surgery (CRS) or palliative surgery [7, 8, 12-15]. CRS + hyperthermic intraoperative peritoneal chemotherapy (HIPEC) is an alternative treatment for these patients. The main principle of CRS+HIPEC is to remove all visible (macroscopic) peritoneal metastases followed by HIPEC to treat any remaining microscopic peritoneal metastases [16]. HIPEC involves peritoneal circulation of chemotherapy drugs (usually mitomycin C, 5-Fluorouracil, and oxaliplatin, or cisplatin) [17] heated to temperatures of 42° C, at which the chemotherapy drugs are potentiated [18]. Until only a decade ago, less than 5% of patients with peritoneal metastases underwent CRS+HIPEC, however this has progressively increased to about 10% of patients by 2012 [8, 9, 14]. CRS+HIPEC has been commissioned by NHS England for patients with peritoneal metastases from appendiceal tumours and colorectal adenocarcinoma.

Why is this research important to patients and health and care services?

Although CRS+HIPEC has the potential to improve the survival and health-related quality of life (HRQoL) in people with peritoneal metastases [14, 19, 20], there have been concerns raised about its

For peer review only

safety. Reports have shown a 30-day mortality after CRS+HIPEC of 1-3% [6], and a major complication rate of 32% [6, 21], albeit that local audit data from high volume centres suggest that mortality and morbidity rates are somewhat less than in these reports (local audit data). The average costs of CRS+HIPEC per patient varies from about 20,000 USD to 80,000 USD [22-28]. Because of these reasons, this research is important to address the significant uncertainty about the benefits of an intervention that carries significant risk of harm to patients and costs to the NHS.

Review of existing evidence

There have been several overviews, systematic reviews, and health technology assessments (HTA) investigating this area. Sixteen systematic reviews of comparative studies have been undertaken, comparing CRS+HIPEC to other treatment modalities in peritoneal metastases from colorectal, ovarian, or gastric cancer [6, 17, 20, 29-41]. Ten of these included at least one RCT, but the conclusions were largely based on non-randomised studies [6, 17, 20, 29, 31-33, 35, 39, 41]. Although most of these systematic reviews concluded that CRS+HIPEC can improve survival in people with peritoneal metastases, all had limitations and deficiencies. Firstly, all are at high risk of bias according to ROBIS (risk of bias in systematic reviews) tool [42] with concern about bias across all domains. Secondly, the systematic reviews included only a single RCT [13] and/or based their evidence predominantly on non-randomised studies, without any adjustment for baseline differences in disease-related or patient-related prognostic characteristics [6, 17, 20, 29, 31-33, 35, 39, 41]. Finally, meta-analyses could only include a small proportion of the results from the studies because of the way these results had been reported (e.g. proportion survived versus median survival) [17, 20, 29, 35, 37]. Therefore, there is still considerable uncertainty about the benefits of CRS+HIPEC and which patient groups will benefit from it.

There have been two formal HTAs on this issue [26, 43]. The HTA reviewing patients with peritoneal disease from colorectal cancer concluded that there was moderate quality evidence that CRS+HIPEC prolonged survival based on a single RCT, but the costs were high [26]. The HTA on

For peer review only

ovarian cancer (which did not include any RCTs) concluded there was no clear benefit of CRS+HIPEC for ovarian peritoneal metastases [43].

Justification for IPD

Through the collection and reanalysis of IPD from all relevant randomised controlled trials, we aim to overcome the limitations of the existing evidence and provide the highest quality evidence synthesis of the benefits and harms of CRS + HIPEC in patients with peritoneal metastases to inform clinical practice and future research. Importantly, the main advantages of using IPD over aggregate data in this setting are the following.

1. Overcome lack of reporting of key survival outcomes: The key survival outcomes have not been reported in a format that can be meta-analysed. This can be overcome with IPD.
2. Harmonise definitions of performance indicators and outcome: Use of IPD can ensure that the definitions of the prognostic and confounding factors, and outcomes are harmonised.
3. Improve the quality of the analysis: IPD is commonly reported to improve the quality of analyses [44, 45].
4. Investigate whether any patient-related or disease-related characteristics impact on the treatment effect at the individual level

Aims and objectives

The overarching aim of this project is to answer the following research question:

Does CRS+HIPEC improve survival and/or quality of life compared to SoC in people with peritoneal metastases (from colorectal, ovarian, or gastric cancers) who can withstand major surgery and is it cost-effective in the NHS setting?

Primary objectives

To compare the relative benefits and harms of CRS+HIPEC versus SoC in people with peritoneal metastases from colorectal, ovarian, or gastric cancers eligible to undergo CRS+HIPEC by a systematic review and IPD meta-analysis.

For peer review only

Secondary objectives

To compare the cost-effectiveness of CRS+HIPEC versus SoC from an NHS and PSS (personal social services) perspective using a model-based cost-utility analysis.

General Methods

Eligibility criteria

Type of studies

All RCTs regardless of the publication status, year of publication, and language of publication will be included.

Setting

Secondary or tertiary care with expertise to perform CRS+HIPEC

Type of participants

Inclusion criteria

People with synchronous or metachronous peritoneal metastases from colorectal cancer, ovarian cancer, or gastric cancer, eligible to undergo CRS+HIPEC regardless of the involvement of other organs and whether the primary cancer was resected completely (i.e. R0 resection). We will also include people with appendiceal adenocarcinomas under colorectal cancer as they behave in a similar way to colorectal adenocarcinomas.

Exclusion criteria

Studies on pseudomyxoma peritonei (PMP) will be excluded.

Intervention

CRS+HIPEC

Control

Standard of care (SoC)

For peer review only

Outcomes

Primary outcomes

1. Overall survival, defined as time from randomisation until death by any cause.
2. HRQoL using any validated measure
3. Serious adverse events or Clavien-Dindo grade III or above [46, 47]

Secondary outcomes

4. Time to disease progression: defined as time from randomisation to death in people who died of treatment or disease-related causes, time from randomisation to recurrence in people in whom complete CRS was achieved, and time from randomisation to disease progression as defined by RECIST (Response Evaluation Criteria in Solid Tumors) criteria of 20% increase in size of the tumour or appearance of new lesions [48], or similar criteria used by authors
5. Non-serious adverse events or Clavien-Dindo grade I or II [46, 47]
6. Patient reported outcome measures

Search strategy

Electronic searches

We will search MEDLINE, EMBASE, Cochrane library, and the Science Citation Index for published trials as well as ClinicalTrials.gov, and WHO ICTRP trial registers for ongoing or unreported studies. The search strategies, which combine the Cochrane sensitivity maximising RCT filter [49] with a combination of subject headings and free text terms relating to the interventions and diseases of interest, are provided in Appendix 1. Searches were updated periodically until October 2019.

Other resources

We will also search the references of all identified studies for additional studies eligible for inclusion. We will also contact the American Society of Peritoneal Surface Malignancies, the Canadian HIPEC

For peer review only

Collaborative Group (CHICG), The Peritoneal Surface Oncology Group International (PSOGI), and the study authors who agree to participate in this project for further studies.

Data collection and management

Selection of studies

Two review authors will independently screen the titles and abstracts of all records retrieved and make the final selection based on full text (after translation if required, i.e. there will be no language restrictions). We will document the process to enable completion of the PRISMA flow-chart. We will resolve discrepancies through discussion and arbitration.

Data collection

At the study level, we will record the contact details of the study author and the study contact, information required to assess the risk of bias, details of the treatment centres (name and the average number of CRS+HIPEC performed per year). At the participant level, we will collect the following details:

1. Centre at which treated
2. Patient demographics: age, gender, comorbidities, performance index
3. Cancer details (including severity)
4. Intervention details
5. Control details
6. Follow-up details
7. Outcome data
8. Resource utilisation data
    a. Operating time
    b. Quantity of blood and blood products transfused
    c. Length of hospital stay (including readmissions)

For peer review only

d. Length of intensive care unit stay

e. Chemotherapy regimen used in HIPEC and in control group if applicable

f. Proportion in whom surgery was performed and the nature of surgery in the control group

g. Additional surgery and other palliative treatments

These data will be sought for all patients randomised into each trial. Up to date follow-up will be requested in order to report on longer-term outcomes: the existing ethical approval for the studies usually cover collection of data.

The proposed data format and coding conventions for these data will be developed as part of the project to obtain the EVERPET-IPD data dictionary. Transfer guide will be developed as part of the project. Although the aim of the conventions is to facilitate data transfer, they are not essential. Data will be accepted in the format most convenient for the individual trial investigator or data centre, however, all personal identifiers (e.g. names) are to be removed before sharing. Data should be transferred by encrypted email or source ftp site. Further details are included in the data transfer guide.

Data checking and management

Once trial investigators have agreed to provide the IPD, they will be asked to sign a data transfer agreement that covers the transfer, use and storage of that data. By signing up to the agreement, investigators also declare that they have complied with all laws and regulations relating to the conduct of their studies and the collection of data as part of those studies.

On receipt, data will be cleaned and checked for accuracy, consistency and validity. This will include checks for missing data, randomisation integrity, follow-up and censoring. We will query any anomalies with the study contact to ensure that the data are represented accurately, and send a summary of the final dataset from each trial to the study contacts for verification.

For peer review only

Once checked and verified, we will store the trial data securely. Access to the data will be restricted to the Project Management Group, who are all trained in data protection and personal data confidentiality and who will act as custodians of the data under the terms of the data transfer agreement, which will be developed as part of this project. In line with that agreement, data will be deposited in the EVidencE Review of PEritoneal Tumours (EVERPET)-IPD repository.

Assessment of risk of bias in included studies

We will use the Cochrane risk of bias tool version 2 to assess the risk of bias in RCTs [50].

Meta-analysis of clinical effectiveness

Measures of treatment effect and data synthesis

We will use risk ratio for binary outcomes (proportion of people with serious and non-serious adverse events), mean difference (if same scales are reported in the studies) or standardised mean difference (if different scales are reported in the studies) for continuous outcomes (HRQoL), rate ratios for count outcomes (number of serious and non-serious adverse events), and hazard ratio for time-to-event outcomes (overall all-cause mortality and time to progression) with their respective 95% confidence intervals.

We will perform a two-step IPD, i.e. calculate the adjusted effect estimate from each included study and then perform a random-effects model meta-analysis using DerSimonian and Laird method [51] for binary outcomes and inverse variance method for other types of outcomes. The reason for choosing the two-step IPD over one-step IPD is the way the confounding factors are reported in the studies, for example, comorbidities can be reported as different types of performance indices and the extent of peritoneal disease can be reported in different ways [52, 53]. However, if we agree on an approximation to convert different performance indices into a single measure and convert the different measures of extent of peritoneal involvement into a single measure, we will perform a single-step meta-analysis to check the robustness of the two-step meta-analysis. We will test our assumptions in approximations (of the different performance indices into a single measure and

For peer review only

different measures of extent of peritoneal involvement into a single measure) by sensitivity analyses. We will use multilevel modelling to take the clustering of data in the studies into account for the one-step IPD meta-analysis, as the unit of analysis will be the individual participant.

Dealing with missing data

We will perform an intention-to-treat analysis whenever possible [54]. If data on the classification of the treatment as intervention or control is missing, and cannot be ascertained though discussion with trialists, we will exclude such participants. If outcome data are missing, we will use multiple imputation method if the data are likely to be missing at random or best-case and worst-case scenarios analysis if it is felt that the outcome data are not missing at random.

Assessment and investigation of heterogeneity

We will assess the clinical and methodological heterogeneity by carefully examining the characteristics and design of included trials. Clinical heterogeneity could be due to the type of participants included in the studies (performance index, stage of cancer, extent of peritoneal involvement, other organ involvement), different interventions (complete CRS or not, chemotherapy agents used), different controls (chemotherapy alone or CRS or both), whether complete CRS was achieved (if the control group was CRS), or different follow-up methods (routine imaging versus clinical examination). Different study designs and risk of bias may contribute to methodological heterogeneity. We will calculate and report the between-trial standard deviation and I^2 as measures of heterogeneity.

If we identify substantial clinical, methodological, or statistical heterogeneity, we will explore and address it in subgroup analyses and/or metaregression using participant level covariates on the sources of clinical heterogeneity mentioned above except for routine imaging which will be a trial-level covariate. All sources of methodological heterogeneity will be trial-level covariates.

Sensitivity analysis

We will perform the following sensitivity analyses to assess the impact of:

For peer review only

- data not missing at random
- non-participation in the IPD
- methods (two-step versus single-step) and model (fixed-effect versus random-effects model) used for meta-analysis
- using 'time from diagnosis' rather than 'time from randomisation' for defining 'time to disease progression'
- risk of bias.

Network meta-analysis

We will also perform a network meta-analysis of aggregate data to compare the three treatments:

1. CRS + HIPEC + systemic chemotherapy
2. CRS alone + systemic chemotherapy
3. Systemic chemotherapy

Reporting bias

We will assess reporting bias by the completeness of search.

Confidence in results

The uncertainty in results will be evaluated using the GRADE methodology [55].

Cost-effectiveness analysis

We will follow the NICE methodological standards for conducting our cost-effectiveness analysis [56].

Model

We will perform a model-based cost-utility analysis estimating mean costs and quality-adjusted life years (QALYs) per patient. We will compare CRS+HIPEC versus SoC in each of the three cancers by three separate cost-effectiveness analyses. The time horizon will be life-time time horizon. We will calculate the costs from the NHS and personal social services (PSS) perspective. We will discount the costs and utilities at the rate of 3.5% per annum [56].

For peer review only

We will create a decision tree model (one for each cancer) along the lines of the model that we used to compare two types of surgeries in pancreatic cancer [57]. Briefly, a patient with peritoneal metastases from one of the three cancers (colorectal cancer, ovarian cancer, or gastric cancer) and eligible for CRS+HIPEC can either undergo CRS+HIPEC or SoC. A proportion of patients undergoing CRS+HIPEC will have complete CRS (i.e. all macroscopic tumour is removed). A proportion of patients in whom CRS+HIPEC will develop complications (whether complete CRS was achieved or not), a proportion of whom may die within 90 days. Those who are alive at 90 days may die subsequently (a Markov model will be used to model this). The decision tree pathways in the people who had SoC will be identical: some will have complete CRS, some will have complications, some will die within 90 days, and some will die after 90 days.

Most of the information required for populating the decision tree (including resource utilisation data) will be obtained from the systematic review and IPD meta-analysis. For information not available from the systematic review and IPD meta-analysis, we will perform literature searches of NHS Economic Evaluation Database (NHS EED), the Health Economic Evaluations Database (HEED), MEDLINE, and EMBASE (for MEDLINE and EMBASE, we will combine the search strategy from Appendix 1 with sensitivity maximising 'economics' filter developed as a part of The Hedges Project of the Health Information Research Unit of McMaster University). We will also review the Cost-Effectiveness Analysis Registry (CEA) at Tufts University for information on quality of life. Currently, there is no HRG (Healthcare Resource Group) code available for CRS+HIPEC and SoC (which will vary according to the nature of the treatment). We will obtain resource utilisation data as part of the systematic review and IPD (please see above) and convert these to costs on the basis of NHS National Tariff, NHS National Schedule of Reference costs, British National Formulary, and/or local estimates as required.

We will assume that the people who die in each period will do so at a constant rate during the period and check whether this assumption is true using the IPD. If this assumption is not true, then we will use more complex models to mirror the survival curves based on the IPD. When no data

For peer review only - http://bmjopen.bmj.com/site/about/guidelines.xhtml

are available from the IPD or published sources, a range of values will be used in the model. We will tabulate the inputs used in the decision tree model and the source of these inputs in the project report.

Measuring cost-effectiveness

We will measure cost-effectiveness using net monetary benefits (NMBs). For each treatment, we will calculate the NMB as the mean QALYs per patient accruing to that treatment multiplied by decision-makers' maximum willingness to pay for a QALY (also referred to as the cost-effectiveness threshold), minus the mean cost per patient for the treatment. In the UK, the upper limit of the maximum willingness to pay for a QALY is £20,000 to £30,000 [56]. NMBs will be calculated using the base case parameter values to obtain the deterministic results, which do not depend on chance. The option with the highest NMB represents best value for money. The NMB for CRS+HIPEC minus the NMB for SoC is the incremental NMB. If the incremental NMB is positive then CRS+HIPEC represents better value for money; if it is negative, the SoC represents better value for money.

A probabilistic sensitivity analysis (PSA) will also be undertaken [56]. The PSA involves Monte Carlo simulation and takes variability of all selected inputs into account simultaneously. Distributions will be assigned to parameters to reflect the uncertainty with each parameter value. A random value from the corresponding distribution for each parameter will be selected (by the computer). This will generate an estimate of the mean cost and mean QALYs and the NMB associated with each treatment. This will be repeated 10,000 times and the results for each simulation will be noted. The mean costs, QALYs and NMB for each treatment will be calculated from the 10,000 simulations; these are probabilistic results because they depend on chance. We will increase the simulations as required by a stability test, which involves ensuring that the standard error when the PSA is repeated 30 times is within 2% of each other. The NMB will also calculated for each of the 10,000 simulations and the proportion of times each treatment had the highest NMB will be calculated for a range of values for the maximum willingness to pay for a QALY. These will be summarised graphically using cost-effectiveness acceptability curves. We will derive the 95% confidence intervals around the base

For peer review only

case values using the 2.5 and 97.5 percentiles calculated from the PSA. We will also perform a value of information analysis and calculate the expected value of perfect information and the expected value of partially perfect information.

For the deterministic univariate sensitivity analysis, each variable in the cost-effectiveness model will be varied one at a time. The results of the sensitivity analysis will be represented in the tornado diagram which reflects the variation in the NMB within the range of the lowest and highest value used for a parameter with all else equal. If the variation in the NMB includes 0, then there is uncertainty in the cost-effectiveness due to the variation of the parameter.

We will also perform various subgroup analyses guided by the results of the systematic reviews and IPD meta-analyses, but will include subgroup analysis of different types of control (i.e. CRS alone or systemic chemotherapy alone or both) as a minimum. We will also perform a sensitivity analysis using information from 'real-life' prospective data from Christie NHS foundation trust (and from other NHS specialist centres if such information is available).

We will follow the 'Consolidated Health Economic Evaluation Reporting Standards' (CHEERS) reporting checklist for reporting the cost-effectiveness analysis [58].

Patient and public involvement

A patient representative was involved in the preparation of this grant proposal and found that this research proposal was important to patients. Additional patient representatives will be identified. A patient representative will also be part of the research oversight committee.

For peer review only

Abbreviations

CEA	Cost-Effectiveness Analysis Registry
CHEERS	Consolidated Health Economic Evaluation Reporting Standards
CHICG	The Canadian HIPEC Collaborative Group
CRS	Cytoreductive surgery
EVERPET	EVidencE Review of PEritoneal Tumours
HEED	Health Economic Evaluations Database
HIPEC	Hyperthermic intraoperative peritoneal chemotherapy
HRG	Healthcare Resource Group
HRQoL	Health-related quality of life
HTA	Health technology assessments
IPD	Individual participant data
NHS	National Health Service
NHS EED	NHS Economic Evaluation Database
NMBs	Net monetary benefits
PMP	Pseudomyxoma peritonei
PRISMA-P	Preferred Reporting Items for Systematic Review and Meta-Analysis Protocols
PSA	Probabilistic sensitivity analysis
PSOGI	The Peritoneal Surface Oncology Group International
PSS	Personal social services
QALYs	Quality-adjusted life years
R0 resection	Resected completely
RCTs	Randomised controlled trials
RECIST	Response Evaluation Criteria in Solid Tumors
ROBIS	Risk of bias in systematic reviews
SoC	Standard of care

For peer review only

Declarations

PROSPERO registration

CRD42019130504

Ethics approval

This project was approved by the UCL Research Ethics Committee (Ethics number: 16023/001).

Consent to participate

Not applicable as this is a systematic review.

Consent for publication

All authors approved this manuscript for publication. There is no other participant information included.

Dissemination and reporting plan

The authorship of the systematic review manuscript will comprise the Project Management Group (the authors of this manuscript), International Advisory Group, representatives from the included trials and patient representatives. We aim to present the findings at appropriate international meetings and publish the review, irrespective of the findings, in a peer-reviewed journal.

Availability of data and materials

Summary data will be shared. Reidentification risk of anonymous data will be assessed at the end of the project and ethical approval will be sought for onward sharing of anonymous data, if the reidentification risk is considered negligible.

Competing interests/Disclosures

The clinical practice of the clinicians in the project: Mr Tim Mould, Mr Muntzer Mughal, Dr Mark Saunders, Mr Omer Aziz, and Prof Sarah O'Dwyer may be altered by the findings of the review.

For peer review only

Funding

This study is funded by the National Institute for Health Research (NIHR) HTA programme (HTA - Project: 17/135/02).

Role of funder: The funder sought independent peer review before funding and approved the protocol. All dissemination must be approved by the funder before dissemination.

Disclaimer: The views expressed are those of the author(s) and not necessarily those of the NIHR or the Department of Health and Social Care.

For peer review only

Sponsor

University College London

Authors contributions

Kurinchi Gurusamy and Claire Vale wrote the manuscript. The manuscript was critically revised by Elena Pizzo, R Bhanot, Brian Davidson, Tim Mould, Muntzer Mughal, Mark Saunders, Omer Aziz, Sarah O'Dwyer. Kurinchi Gurusamy is the guarantor of this manuscript.

Acknowledgements

We used the PRISMA-P checklist when writing our report ('Moher D, Shamseer L, Clarke M, Ghersi D, Liberati A, Petticrew M, Shekelle P, Stewart LA. Preferred Reporting Items for Systematic Review and Meta-Analysis Protocols (PRISMA-P) 2015 statement. Syst Rev. 2015;4(1):1').

Licence

I, the Submitting Author has the right to grant and does grant on behalf of all authors of the Work (as defined in the below author licence), an exclusive licence on a worldwide, perpetual, irrevocable, royalty-free basis to BMJ Publishing Group Ltd ("BMJ") its licensees and where the relevant Journal is co-owned by BMJ to the co-owners of the Journal, to publish the Work in BMJ Open and any other BMJ products and to exploit all rights, as set out in our licence. The Submitting Author accepts and understands that any supply made under these terms is made by BMJ to the Submitting Author.

References

1. International Agency for Research on Cancer (World Health Organization): **Estimated number of incident cases, both sexes, all cancers excluding non-melanoma skin cancer, worldwide in 2012**. https://gco.iarc.fr/today/online-analysis-table?mode=cancer&mode_population=continents&population=900&sex=0&cancer=29&type=0&statistic=0&prevalence=0&color_palette=default *(accessed on 3rd December 2017)* 2017.

2. Lemmens VE, Klaver YL, Verwaal VJ, Rutten HJ, Coebergh JWW, de Hingh IH: **Predictors and survival of synchronous peritoneal carcinomatosis of colorectal origin: A population-based study**. *International Journal of Cancer* 2011, **128**(11):2717-2725.

3. Riihimäki M, Hemminki A, Sundquist J, Hemminki K: **Patterns of metastasis in colon and rectal cancer**. *Scientific Reports* 2016, **6**:29765.

4. Segelman J, Granath F, Holm T, Machado M, Mahteme H, Martling A: **Incidence, prevalence and risk factors for peritoneal carcinomatosis from colorectal cancer**. *British Journal of Surgery* 2012, **99**(5):699-705.

5. van Gestel YRBM, Thomassen I, Lemmens VEPP, Pruijt JFM, van Herk-Sukel MPP, Rutten HJT, Creemers GJ, de Hingh IHJT: **Metachronous peritoneal carcinomatosis after curative treatment of colorectal cancer**. *European Journal of Surgical Oncology (EJSO)* 2014, **40**(8):963-969.

6. Baratti D, Kusamura S, Pietrantonio F, Guaglio M, Niger M, Deraco M: **Progress in treatments for colorectal cancer peritoneal metastases during the years 2010-2015. A systematic review**. *Critical Reviews in Oncology/Hematology* 2016, **100**:209-222.

7. Thomassen I, van Gestel YR, van Ramshorst B, Luyer MD, Bosscha K, Nienhuijs SW, Lemmens VE, de Hingh IH: **Peritoneal carcinomatosis of gastric origin: A population-based study on incidence, survival and risk factors**. *International Journal of Cancer* 2014, **134**(3):622-628.

8. Quere P, Facy O, Manfredi S, Jooste V, Faivre J, Lepage C, Bouvier AM: **Epidemiology, Management, and Survival of Peritoneal Carcinomatosis from Colorectal Cancer: A Population-Based Study**. *Dis Colon Rectum* 2015, **58**(8):743-752.

For peer review only

9. Klaver YL, Lemmens VE, Creemers GJ, Rutten HJ, Nienhuijs SW, de Hingh IH: **Population-based survival of patients with peritoneal carcinomatosis from colorectal origin in the era of increasing use of palliative chemotherapy**. *Ann Oncol* 2011, **22**(10):2250-2256.

10. Hwang M, Jayakrishnan TT, Green DE, George B, Thomas JP, Groeschl RT, Erickson B, Pappas SG, Gamblin TC, Turaga KK: **Systematic review of outcomes of patients undergoing resection for colorectal liver metastases in the setting of extra hepatic disease**. *Eur J Cancer* 2014, **50**(10):1747-1757.

11. Spiliotis J, Halkia E, Lianos E, Kalantzi N, Grivas A, Efstathiou E, Giassas S: **Cytoreductive surgery and HIPEC in recurrent epithelial ovarian cancer: a prospective randomized phase III study**. *Annals of surgical oncology* 2015, **22**(5):1570-1575.

12. Yang XJ, Huang CQ, Suo T, Mei LJ, Yang GL, Cheng FL, Zhou YF, Xiong B, Yonemura Y, Li Y: **Cytoreductive surgery and hyperthermic intraperitoneal chemotherapy improves survival of patients with peritoneal carcinomatosis from gastric cancer: Final results of a phase III randomized clinical trial**. *Annals of surgical oncology* 2011, **18**(6):1575-1581.

13. Verwaal VJ, Van Ruth S, De Bree E, Van Slooten GW, Van Tinteren H, Boot H, Zoetmulder FAN: **Randomized trial of cytoreduction and hyperthermic intraperitoneal chemotherapy versus systemic chemotherapy and palliative surgery in patients with peritoneal carcinomatosis of colorectal cancer**. *Journal of Clinical Oncology* 2003, **21**(20):3737-3743.

14. Razenberg LG, van Gestel YR, Creemers GJ, Verwaal VJ, Lemmens VE, de Hingh IH: **Trends in cytoreductive surgery and hyperthermic intraperitoneal chemotherapy for the treatment of synchronous peritoneal carcinomatosis of colorectal origin in the Netherlands**. *Eur J Surg Oncol* 2015, **41**(4):466-471.

15. Verwaal VJ, Bruin S, Boot H, van Slooten G, van Tinteren H: **8-year follow-up of randomized trial: cytoreduction and hyperthermic intraperitoneal chemotherapy versus systemic chemotherapy in patients with peritoneal carcinomatosis of colorectal cancer**. *Annals of surgical oncology* 2008, **15**(9):2426-2432.

For peer review only

16. Elias D, Goere D, Dumont F, Honore C, Dartigues P, Stoclin A, Malka D, Boige V, Ducreux M: **Role of hyperthermic intraoperative peritoneal chemotherapy in the management of peritoneal metastases**. *Eur J Cancer* 2014, **50**(2):332-340.

17. Desiderio J, Chao J, Melstrom L, Warner S, Tozzi F, Fong Y, Parisi A, Woo Y: **The 30-year experience-A meta-analysis of randomised and high-quality non-randomised studies of hyperthermic intraperitoneal chemotherapy in the treatment of gastric cancer**. *European Journal of Cancer* 2017, **79**:1-14.

18. Schaaf L, van der Kuip H, Zopf W, Winter S, Munch M, Murdter TE, Thon KP, Steurer W, Aulitzky WE, Ulmer C: **A Temperature of 40 degrees C Appears to be a Critical Threshold for Potentiating Cytotoxic Chemotherapy In Vitro and in Peritoneal Carcinomatosis Patients Undergoing HIPEC**. *Ann Surg Oncol* 2015, **22**:S758-S765.

19. Seretis C, Youssef H: **Quality of life after cytoreductive surgery and intraoperative hyperthermic intraperitoneal chemotherapy for peritoneal surface malignancies: a systematic review**. *Eur J Surg Oncol* 2014, **40**(12):1605-1613.

20. Huang CQ, Min Y, Wang SY, Yang XJ, Liu Y, Xiong B, Yonemura Y, Li Y: **Cytoreductive surgery plus hyperthermic intraperitoneal chemotherapy improves survival for peritoneal carcinomatosis from colorectal cancer: A systematic review and meta-analysis of current evidence**. *Oncotarget* 2017, **8**(33):55657-55683.

21. Cavaliere D, Cirocchi R, Coccolini F, Fagotti A, Fambrini M, Federici O, Lorusso D, Vaira M, Ceresoli M, Delrio P *et al*: **1st Evidence-based Italian consensus conference on cytoreductive surgery and hyperthermic intraperitoneal chemotherapy for peritoneal carcinosis from ovarian cancer**. *Tumori* 2017, **103**(6):525-536.

22. Baratti D, Scivales A, Balestra MR, Ponzi P, Di Stasi F, Kusamura S, Laterza B, Deraco M: **Cost analysis of the combined procedure of cytoreductive surgery and hyperthermic intraperitoneal chemotherapy (HIPEC)**. *Ejso* 2010, **36**(5):463-469.

For peer review only

23. Bonastre J, Chevalier J, Elias D, Classe JM, Ferron G, Guilloit JM, Marchal F, Meeus P, De Pouvourville G: **Cost-effectiveness of intraperitoneal chemohyperthermia in the treatment of peritoneal carcinomatosis from colorectal cancer**. *Value in Health* 2008, **11**(3):347-353.

24. Chua TC, Martin S, Saxena A, Liauw W, Yan TD, Zhao J, Lok I, Morris DL: **Evaluation of the cost-effectiveness of cytoreductive surgery and hyperthermic intraperitoneal chemotherapy (peritonectomy) at the st george hospital peritoneal surface malignancy program**. *Annals of Surgery* 2010, **251**(2):323-329.

25. Lee Z, Chia C, Teo M: **Is cytoreductive surgery and hyperthermic intraperitoneal chemotherapy cost-effective for metastatic colorectal cancer?** *Annals of surgical oncology* 2016, **23**(1 Suppl):S77.

26. Ludwigs K, Breimer ME, Brorson F, Carlsson G, Daxberg EL, Hjalmarsson Y, Skullman S, Strandell A, Jivegård L: **Cytoreductive surgery and intraperitoneal chemotherapy (HIPEC or EPIC) in patients with colorectal adenocarcinoma and peritoneal carcinomatosis. (Structured abstract)**. *Health Technology Assessment Database* 2013(4).

27. Naffouje SA, O'Donoghue C, Salti GI: **Evaluation of cytoreductive surgery and hyperthermic intraperitoneal chemotherapy in a community setting: A cost-utility analysis of a hospital's initial experience and reflections on the health care system**. *Journal of Surgical Oncology* 2016, **113**(5):544-547.

28. Tentes AAK, Pallas N, Korakianitis O, Mavroudis C, Spiridonidou A, Zorbas G, Popidis S, Papadoniou N, Darladima V, Smyrnis A *et al*: **The cost of cytoreductive surgery and perioperative intraperitoneal chemotherapy in the treatment of peritoneal malignancy in one Greek institute**. *Journal of B.U.ON.* 2012, **17**(4):776-780.

29. Cao C, Yan TD, Black D, Morris DL: **A systematic review and meta-analysis of cytoreductive surgery with perioperative intraperitoneal chemotherapy for peritoneal carcinomatosis of colorectal origin**. *Annals of surgical oncology* 2009, **16**(8):2152-2165.

For peer review only

30. Ceelen WP, Hesse U, de Hemptinne B, Pattyn P: **Hyperthermic intraperitoneal chemoperfusion in the treatment of locally advanced intra-abdominal cancer**. *British Journal of Surgery* 2000, **87**(8):1006-1015.

31. Chia CS, Seshadri RA, Kepenekian V, Vaudoyer D, Passot G, Glehen O: **Survival outcomes after Cytoreductive surgery and hyperthermic intraperitoneal chemotherapy for peritoneal carcinomatosis from gastric cancer: A systematic review**. *Pleura and Peritoneum* 2016, **1**(2):67-77.

32. Chua TC, Esquivel J, Pelz JO, Morris DL: **Summary of current therapeutic options for peritoneal metastases from colorectal cancer**. *Journal of Surgical Oncology* 2013, **107**(6):566-573.

33. Di Vita M, Cappellani A, Piccolo G, Zanghi A, Cavallaro A, Bertola G, Bolognese A, Facchini G, D'Aniello C, Di Francia R *et al*: **The role of HIPEC in the treatment of peritoneal carcinomatosis from gastric cancer: Between lights and shadows**. *Anti-Cancer Drugs* 2015, **26**(2):123-138.

34. Eveno C, Pocard M: **Randomized controlled trials evaluating Cytoreductive surgery (CRS) and hyperthermic intraperitoneal chemotherapy (HIPEC) in prevention and therapy of peritoneal metastasis: A Systematic review**. *Pleura and Peritoneum* 2016, **1**(4):169-182.

35. He TF, Chen ZR, Xing CG: **Cytoreductive surgery combined with intraperitoneal chemotherapy in the treatment of colorectal peritoneal metastasis: a meta-analysis**. *International Journal of Clinical and Experimental Medicine* 2016, **9**(11):20562-20570.

36. Hotouras A, Desai D, Bhan C, Murphy J, Lampe B, Sugarbaker PH: **Heated IntraPEritoneal Chemotherapy (HIPEC) for patients with recurrent ovarian cancer: A systematic literature review**. *International Journal of Gynecological Cancer* 2016, **26**(4):661-670.

37. Huo YR, Richards A, Liauw W, Morris DL: **Hyperthermic intraperitoneal chemotherapy (HIPEC) and cytoreductive surgery (CRS) in ovarian cancer: A systematic review and meta-analysis**. *European Journal of Surgical Oncology* 2015, **41**(12):1578-1589.

38. Lopez-Lopez V, Cascales-Campos PA, Schneider MA, Gil J, Gil E, Gomez-Hidalgo NR, Parrilla P: **Cytoreductive surgery and hyperthermic intraperitoneal chemotherapy (HIPEC) in elderly patients. A systematic literature review**. *Surgical Oncology-Oxford* 2016, **25**(4):378-384.

For peer review only

39. Mirnezami R, Moran BJ, Harvey K, Cecil T, Chandrakumaran K, Carr N, Mohamed F, Mirnezami AH: **Cytoreductive surgery and intraperitoneal chemotherapy for colorectal peritoneal metastases**. *World Journal of Gastroenterology* 2014, **20**(38):14018-14032.

40. Kireeva GS, Gafton GI, Guseynov KD, Senchik KY, Belyaeva OA, Bespalov VG, Panchenko AV, Maydin MA, Belyaev AM: **HIPEC in patients with primary advanced ovarian cancer: Is there a role? A systematic review of short- and long-term outcomes**. *Surg Oncol* 2018, **27**(2):251-258.

41. Morano WF, Khalili M, Chi DS, Bowne WB, Esquivel J: **Clinical studies in CRS and HIPEC: Trials, tribulations, and future directions-A systematic review**. *J Surg Oncol* 2018, **117**(2):245-259.

42. Whiting P, Savovic J, Higgins JP, Caldwell DM, Reeves BC, Shea B, Davies P, Kleijnen J, Churchill R, group R: **ROBIS: A new tool to assess risk of bias in systematic reviews was developed**. *J Clin Epidemiol* 2016, **69**:225-234.

43. Ubago-Pérez R, Matas-Hoces A, Beltrán-Calvo C, Romero-Tabares A: **Hyperthermic intraperitoneal chemotherapy. Efficacy and safety in the treatment of ovarian cancer peritoneal carcino matosis (Structured abstract)**. *Health Technology Assessment Database* 2013(4).

44. Riley RD, Lambert PC, Abo-Zaid G: **Meta-analysis of individual participant data: rationale, conduct, and reporting**. *BMJ* 2010, **340**.

45. Tierney JF, Vale C, Riley R, Smith CT, Stewart L, Clarke M, Rovers M: **Individual Participant Data (IPD) Meta-analyses of Randomised Controlled Trials: Guidance on Their Use**. *PLoS Med* 2015, **12**(7):e1001855.

46. Clavien PA, Barkun J, de Oliveira ML, Vauthey JN, Dindo D, Schulick RD, de Santibanes E, Pekolj J, Slankamenac K, Bassi C *et al*: **The Clavien-Dindo classification of surgical complications: five-year experience**. *Ann Surg* 2009, **250**(2):187-196.

47. Dindo D, Demartines N, Clavien PA: **Classification of surgical complications: a new proposal with evaluation in a cohort of 6336 patients and results of a survey**. *Ann Surg* 2004, **240**(2):205-213.

48. Therasse P, Arbuck SG, Eisenhauer EA, Wanders J, Kaplan RS, Rubinstein L, Verweij J, Van Glabbeke M, van Oosterom AT, Christian MC *et al*: **New guidelines to evaluate the response to**

For peer review only

treatment in solid tumors. European Organization for Research and Treatment of Cancer, National Cancer Institute of the United States, National Cancer Institute of Canada. *J Natl Cancer Inst* 2000, **92**(3):205-216.

49. Higgins J, Green S, (editors): **Cochrane Handbook for Systematic Reviews of Interventions Version 5.1.0 [updated March 2011]**. *The Cochrane Collaboration. Available from* *www.handbook.cochrane.org* 2011.

50. Sterne JAC, Savović J, Page MJ, Elbers RG, Blencowe NS, Boutron I, Cates CJ, Cheng H-Y, Corbett MS, Eldridge SM *et al*: **RoB 2: a revised tool for assessing risk of bias in randomised trials**. *BMJ* 2019, **366**:l4898.

51. DerSimonian R, Laird N: **Meta-analysis in clinical trials**. *Control Clin Trials* 1986, **7**(3):177-188.

52. Ng JL, Ong WS, Chia CS, Tan GH, Soo KC, Teo MC: **Prognostic Relevance of the Peritoneal Surface Disease Severity Score Compared to the Peritoneal Cancer Index for Colorectal Peritoneal Carcinomatosis**. *Int J Surg Oncol* 2016, **2016**:2495131.

53. Esquivel J, Lowy AM, Markman M, Chua T, Pelz J, Baratti D, Baumgartner JM, Berri R, Bretcha-Boix P, Deraco M *et al*: **The American Society of Peritoneal Surface Malignancies (ASPSM) Multiinstitution Evaluation of the Peritoneal Surface Disease Severity Score (PSDSS) in 1,013 Patients with Colorectal Cancer with Peritoneal Carcinomatosis**. *Annals of surgical oncology* 2014, **21**(13):4195-4201.

54. Newell DJ: **Intention-to-treat analysis: implications for quantitative and qualitative research**. *International journal of epidemiology* 1992, **21**(5):837-841.

55. Guyatt G, Oxman AD, Akl EA, Kunz R, Vist G, Brozek J, Norris S, Falck-Ytter Y, Glasziou P, DeBeer H *et al*: **GRADE guidelines: 1. Introduction-GRADE evidence profiles and summary of findings tables**. *J Clin Epidemiol* 2011, **64**(4):383-394.

56. National Institute for Health and Care Excellence: **Guide to the methods of technology appraisal 2013**. *https://www.nice.org.uk/process/pmg9/chapter/foreword (accessed on 4th June 2018)* 2013.

For peer review only

57. Gurusamy KS, Riviere D, van Laarhoven CJH, Besselink M, Abu-Hilal M, Davidson BR, Morris S: **Cost-effectiveness of laparoscopic versus open distal pancreatectomy for pancreatic cancer**. *PLoS One* 2017, **12**(12):e0189631.

58. Husereau D, Drummond M, Petrou S, Carswell C, Moher D, Greenberg D, Augustovski F, Briggs AH, Mauskopf J, Loder E *et al*: **Consolidated Health Economic Evaluation Reporting Standards (CHEERS)--explanation and elaboration: a report of the ISPOR Health Economic Evaluation Publication Guidelines Good Reporting Practices Task Force**. *Value Health* 2013, **16**(2):231-250.

For peer review only

Appendix 1: Search Strategies

Medline

1. Hyperthermia, Induced/

2. ((hyperthermic or heated) adj3 (intraperitoneal or intra-peritoneal) adj3 (chemotherapy or chemotherapies)).ti,ab.

3. (intraperitoneal adj3 chemohyperthermia).ti,ab.

4. (HIPEC or IPHC or HIIC).ti,ab.

5. 1 or 2 or 3 or 4

6. Cytoreduction Surgical Procedures/

7. ((cytoreductive or cytoreduction or debulking) adj3 (surgery or surgeries or surgical or procedure or procedures)).ti,ab.

8. 6 or 7

9. 5 or 8

10. exp Colorectal Neoplasms/

11. exp Ovarian Neoplasms/

12. Stomach Neoplasms/

13. ((colorectal or bowel or colon or colonic or rectum or rectal or ovary or ovaries or ovarian or gastric or stomach) adj3 (cancer or cancers or carcinoma or carcinomas or tumour or tumours or tumor or tumors or neoplasm or neoplasms)).ti,ab.

14. 10 or 11 or 12 or 13

15. 9 and 14

16. randomized controlled trial.pt.

17. controlled clinical trial.pt.

18. randomized.ab.

19. placebo.ab.

For peer review only

20. drug therapy.fs.

21. randomly.ab.

22. trial.ab.

23. groups.ab.

24. 16 or 17 or 18 or 19 or 20 or 21 or 22 or 23

25. exp animals/ not humans.sh.

26. 24 not 25

27. 15 and 26

28. (cost: or cost benefit analys: or health care costs).mp.

29. 15 and 28

30. 27 or 29

Embase

1. hyperthermic intraperitoneal chemotherapy/

2. ((hyperthermic or heated) adj3 (intraperitoneal or intra-peritoneal) adj3 (chemotherapy or chemotherapies)).ti,ab.

3. (intraperitoneal adj3 chemohyperthermia).ti,ab.

4. (HIPEC or IPHC or HIIC).ti,ab.

5. 1 or 2 or 3 or 4

6. cytoreductive surgery/

7. ((cytoreductive or cytoreduction or debulking) adj3 (surgery or surgeries or surgical or procedure or procedures)).ti,ab.

8. 6 or 7

9. 5 or 8

10. exp colon cancer/

11. exp rectum cancer/

12. exp ovary cancer/

For peer review only

13. exp stomach cancer/

14. ((colorectal or bowel or colon or colonic or rectum or rectal or ovary or ovaries or ovarian or gastric or stomach) adj3 (cancer or cancers or carcinoma or carcinomas or tumour or tumours or tumor or tumors or neoplasm or neoplasms)).ti,ab.

15. 10 or 11 or 12 or 13 or 14

16. 9 and 15

17. exp crossover-procedure/ or exp double-blind procedure/ or exp randomized controlled trial/ or single-blind procedure/

18. (((((random* or factorial* or crossover* or cross over* or cross-over* or placebo* or double*) adj blind*) or single*) adj blind*) or assign* or allocat* or volunteer*).af.

19. 17 or 18

20. 16 and 19

21. (cost or costs).tw.

22. 16 and 21

23. 20 or 22

Cochrane

#1 MeSH descriptor: [Hyperthermia, Induced] this term only

#2 ((hyperthermic or heated) near/3 (intraperitoneal or intra-peritoneal) near/3 (chemotherapy or chemotherapies))

#3 (intraperitoneal near/3 chemohyperthermia)

#4 (HIPEC or IPHC or HIIC)

#5 #1 or #2 or #3 or #4

#6 MeSH descriptor: [Cytoreduction Surgical Procedures] this term only

#7 ((cytoreductive or cytoreduction or debulking) near/3 (surgery or surgeries or surgical or procedure or procedures))

#8 #6 or #7

For peer review only

#9 #5 or #8

#10 MeSH descriptor: [Colorectal Neoplasms] explode all trees

#11 MeSH descriptor: [Ovarian Neoplasms] explode all trees

#12 MeSH descriptor: [Stomach Neoplasms] this term only

#13 ((colorectal or bowel or colon or colonic or rectum or rectal or ovary or ovaries or ovarian or gastric or stomach) near/3 (cancer or cancers or carcinoma or carcinomas or tumour or tumours or tumor or tumors or neoplasm or neoplasms))

#14 #10 or #11 or #12 or #13

#15 #9 and #14

Science Citation Index

1 TS=((hyperthermic or heated) near/3 (intraperitoneal or intra-peritoneal) near/3 (chemotherapy or chemotherapies))

2 TS=(intraperitoneal near/3 chemohyperthermia)

3 TS=(HIPEC or IPHC or HIIC)

4 #3 OR #2 OR #1

5 TS=((cytoreductive or cytoreduction or debulking) near/3 (surgery or surger-ies or surgical or procedure or procedures))

6 #5 or #4

7 TS=((colorectal or bowel or colon or colonic or rectum or rectal or ovary or ovaries or ovarian or gastric or stomach) near/3 (cancer or cancers or carci-noma or carcinomas or tumour or tumours or tumor or tumors or neoplasm or neoplasms))

#8 TS=(random* or placebo* or blind* or meta-analysis or cost or costs)

#9 #8 AND #7 AND #6

For peer review only

WHO trials register

Condition: colorectal OR bowel OR colon OR colonic OR rectum OR rectal OR ovary OR ovaries OR ovarian OR gastric OR stomach

Intervention: HIPEC OR hyperthermic intraperitoneal chemotherapy OR IPHC OR intraperitoneal chemohyperthermia OR HIIC OR heated intraoperative intraperitoneal chemotherapy OR cytoreductive surgery OR CRS

ClinicalTrials.gov

Condition: colorectal OR bowel OR colon OR colonic OR rectum OR rectal OR ovary OR ovaries OR ovarian OR gastric OR stomach

Study Type: Interventional Studies (Clinical Trials)

Intervention/treatment: HIPEC OR hyperthermic intraperitoneal chemotherapy OR IPHC OR intraperitoneal chemohyperthermia OR HIIC OR heated intraoperative intraperitoneal chemotherapy OR cytoreductive surgery OR CRS

Interventional studies, phase 2,3,4

Interventional Studies | colorectal OR bowel OR colon OR colonic OR rectum OR rectal OR ovary OR ovaries OR ovarian OR gastric OR stomach | HIPEC OR hyperthermic intraperitoneal chemotherapy OR IPHC OR intraperitoneal chemohyperthermia OR HIIC OR heated intraoperative intraperitoneal chemotherapy OR cytoreductive surgery OR CRS | Phase 2, 3, 4

Cost-Effectiveness Analysis (CEA) Registry

The following terms were searched:

Hyperthermic

Cytoreduction

Cytoreductive

For peer review only

Reporting checklist for protocol of a systematic review.

Based on the PRISMA-P guidelines.

Instructions to authors

Complete this checklist by entering the page numbers from your manuscript where readers will find each of the items listed below.

Your article may not currently address all the items on the checklist. Please modify your text to include the missing information. If you are certain that an item does not apply, please write "n/a" and provide a short explanation.

Upload your completed checklist as an extra file when you submit to a journal.

In your methods section, say that you used the PRISMA-Preporting guidelines, and cite them as:

Moher D, Shamseer L, Clarke M, Ghersi D, Liberati A, Petticrew M, Shekelle P, Stewart LA. Preferred Reporting Items for Systematic Review and Meta-Analysis Protocols (PRISMA-P) 2015 statement. Syst Rev. 2015;4(1):1.

		Reporting Item	Page Number
**Title**			
Identification	#1a	Identify the report as a protocol of a systematic review	1
Update	#1b	If the protocol is for an update of a previous systematic review, identify as such	NA

For peer review only

**Registration**			
	#2	If registered, provide the name of the registry (such as PROSPERO) and registration number	3
**Authors**			
Contact	#3a	Provide name, institutional affiliation, e-mail address of all protocol authors; provide physical mailing address of corresponding author	1-2
Contribution	#3b	Describe contributions of protocol authors and identify the guarantor of the review	3
**Amendments**			
	#4	If the protocol represents an amendment of a previously completed or published protocol, identify as such and list changes; otherwise, state plan for documenting important protocol amendments	NA
**Support**			
Sources	#5a	Indicate sources of financial or other support for the review	3
Sponsor	#5b	Provide name for the review funder and / or sponsor	3
Role of sponsor or funder	#5c	Describe roles of funder(s), sponsor(s), and / or institution(s), if any, in developing the protocol	3
**Introduction**			
Rationale	#6	Describe the rationale for the review in the context of what	6-8

For peer review only

		is already known	
Objectives	#7	Provide an explicit statement of the question(s) the review will address with reference to participants, interventions, comparators, and outcomes (PICO)	8-9
**Methods**			
Eligibility criteria	#8	Specify the study characteristics (such as PICO, study design, setting, time frame) and report characteristics (such as years considered, language, publication status) to be used as criteria for eligibility for the review	9-10
Information sources	#9	Describe all intended information sources (such as electronic databases, contact with study authors, trial registers or other grey literature sources) with planned dates of coverage	10-11
Search strategy	#10	Present draft of search strategy to be used for at least one electronic database, including planned limits, such that it could be repeated	Supplement 1-5
Study records - data management	#11a	Describe the mechanism(s) that will be used to manage records and data throughout the review	11-13
Study records - selection process	#11b	State the process that will be used for selecting studies (such as two independent reviewers) through each phase of the review (that is, screening, eligibility and inclusion in meta-analysis)	11
Study records -	#11c	Describe planned method of extracting data from reports	11-13

For peer review only

data collection process		(such as piloting forms, done independently, in duplicate), any processes for obtaining and confirming data from investigators	
Data items	#12	List and define all variables for which data will be sought (such as PICO items, funding sources), any pre-planned data assumptions and simplifications	11-13
Outcomes and prioritization	#13	List and define all outcomes for which data will be sought, including prioritization of main and additional outcomes, with rationale	10
Risk of bias in individual studies	#14	Describe anticipated methods for assessing risk of bias of individual studies, including whether this will be done at the outcome or study level, or both; state how this information will be used in data synthesis	13
Data synthesis	#15a	Describe criteria under which study data will be quantitatively synthesised	13-14
Data synthesis	#15b	If data are appropriate for quantitative synthesis, describe planned summary measures, methods of handling data and methods of combining data from studies, including any planned exploration of consistency (such as I2, Kendall's τ)	13-14
Data synthesis	#15c	Describe any proposed additional analyses (such as sensitivity or subgroup analyses, meta-regression)	14-15
Data synthesis	#15d	If quantitative synthesis is not appropriate, describe the type of summary planned	NA

For peer review only

Meta-bias(es)	#16	Specify any planned assessment of meta-bias(es) (such as publication bias across studies, selective reporting within studies)	15
Confidence in cumulative evidence	#17	Describe how the strength of the body of evidence will be assessed (such as GRADE)	NA

The PRISMA-P checklist is distributed under the terms of the Creative Commons Attribution License CC-BY 4.0. This checklist was completed on 10. April 2020 using https://www.goodreports.org/, a tool made by the EQUATOR Network in collaboration with Penelope.ai

For peer review only
