# Supplementary material for: Cytoreductive surgery (CRS) with hyperthermic intraoperative peritoneal chemotherapy (HIPEC) versus standard of care (SoC) in people with peritoneal metastases from colorectal, ovarian or gastric origin: protocol for a systematic review and individual participant data (IPD) meta-analyses of effectiveness and cost-effectiveness
Source: BMJ Open. 2020 May 12;10(5):e039314. doi: 10.1136/bmjopen-2020-039314 (PMC7228534; doi:10.1136/bmjopen-2020-039314)
Supplement: Reviewer comments [file bmjopen-2020-039314.reviewer_comments.pdf]

PEER REVIEW HISTORY

BMJ Open publishes all reviews undertaken for accepted manuscripts. Reviewers are asked to complete a checklist review form (**http://bmjopen.bmj.com/site/about/resources/checklist.pdf**) and are provided with free text boxes to elaborate on their assessment. These free text comments are reproduced below.

ARTICLE DETAILS

**TITLE (PROVISIONAL)**	Cytoreductive surgery (CRS) with hyperthermic intraoperative peritoneal chemotherapy (HIPEC) versus standard of care (SoC) in people with peritoneal metastases from colorectal, ovarian or gastric origin: protocol for a systematic review and individual participant data (IPD) meta-analyses of effectiveness and cost-effectiveness
**AUTHORS**	Gurusamy, Kurinchi; Vale, Claire; Pizzo, Elena; Bhanot, R; Davidson, Brian; Mould, Tim; Mughal, Muntzer; Saunders, Mark; Aziz, Omer; O'Dwyer, Sarah

This article was not externally reviewed at BMJ Open. Protocols that have been independently assessed prior to submission to BMJ Open are usually fast-tracked to publication on the grounds that further substantial changes will not be possible. This independent assessment will usually be external, independent review for both a substantial grant award from a non-commercial or government funder and ethics approval.
